# Muscle to Brain Partitioning as Measure of Transporter-Mediated Efflux at the Rat Blood–Brain Barrier and Its Implementation into Compound Optimization in Drug Discovery

**DOI:** 10.3390/pharmaceutics11110595

**Published:** 2019-11-11

**Authors:** Yunhai Cui, Ralf Lotz, Hermann Rapp, Klaus Klinder, Anneke Himstedt, Achim Sauer

**Affiliations:** 1 Department of Drug Discovery Sciences, Boehringer Ingelheim Pharma GmbH & Co. KG, 88397 Biberach, Germany; yunhai.cui@boehringer-ingelheim.com (Y.C.); hermann.rapp@boehringer-ingelheim.com (H.R.); klaus.klinder@boehringer-ingelheim.com (K.K.); anneke.himstedt@uni-hamburg.de (A.H.); 2 Department of Drug Metabolism and Pharmacokinetics, Boehringer Ingelheim Pharma GmbH & Co. KG, 88397 Biberach, Germany; ralf.lotz@boehringer-ingelheim.com; 3 Department of Clinical Pharmacy, Institute of Pharmacy, University of Hamburg, 20146 Hamburg, Germany

**Keywords:** blood–brain barrier, P-gp, BCRP, efflux, K_p,uu,brain_, drug partitioning, CNS drug, screening, drug discovery, in vitro–in vivo correlation

## Abstract

Movement of xenobiotic substances across the blood–brain barrier (BBB) is tightly regulated by various transporter proteins, especially the efflux transporters P-glycoprotein (P-gp/MDR1) and breast cancer resistance protein (BCRP). Avoiding drug efflux at the BBB is a unique challenge for the development of new central nervous system (CNS) drugs. Drug efflux at the BBB is described by the partition coefficient of unbound drug between brain and plasma (K_p,uu,brain)_ which is typically obtained from in vivo and often additionally in vitro measurements. Here, we describe a new method for the rapid estimation of the in vivo drug efflux at the BBB of rats: the measurement of the partition coefficient of a drug between brain and skeletal muscle (K_p,brain/muscle_). Assuming a closely similar distribution of drugs into the brain and muscle and that the efflux transporters are only expressed in the brain, K_p,brain/muscle_, similar to K_p,uu,brain_, reflects the efflux at the BBB. The new method requires a single in vivo experiment. For 64 compounds from different research programs, we show the comparability to other approaches used to obtain K_p,uu,brain_. P-gp- and BCRP-overexpressing cell systems are valuable in vitro tools for prescreening. Drug efflux at the BBB can be most accurately predicted based on a simple algorithm incorporating data from both in vitro assays. In conclusion, the combined use of our new in vivo method and the in vitro tools allows an efficient screening method in drug discovery with respect to efflux at the BBB.

## 1. Introduction

The blood–brain barrier (BBB), formed by endothelial cells of the brain capillaries, astrocytes, and pericytes, is a regulatory interface between the central nervous system (CNS) and the circulatory bloodstream [1]. Movement of endogenous and xenobiotic substances across the BBB is tightly regulated by various transporter proteins expressed in the endothelial cells. On the one hand, it is a unique challenge to develop drugs which can overcome the BBB and reach their pharmacological targets within the CNS. On the other hand, it can also be important to prevent drugs with peripheral targets from entering the CNS due to their potential undesired side effects.

Two membrane transporters have been identified as the major players regulating the transport of drugs across the BBB: multidrug resistance 1 (MDR1)/P-glycoprotein (P-gp)/ATP-binding cassette sub-family B member 1 (ABCB1) and breast cancer resistance protein (BCRP)/ATP-binding cassette super-family G member 2 (ABCG2) [2,3]. Both transporters belong to the ATP-binding cassette (ABC) superfamily of transporters and are localized in the luminal membrane of the brain capillary endothelial cells. Due to their very broad substrate spectrum both transporters can efficiently limit the entry of drugs into brain and thus are considered as major hurdles in the discovery of CNS drugs. There is, therefore, a strong interest to evaluate the impact of both transporters on the brain distribution of drug candidates early in the drug discovery process. In recent years, the partition coefficient of unbound drug between brain and plasma (K_p,uu,brain_) has become more and more relevant for the description of the extent of brain penetration of a drug [4,5]. Under steady-state conditions, K_p,uu,brain_ defines the ratio between the unbound drug concentration in brain (C_u,brain_), i.e., the interstitial fluid (ISF) and the unbound drug concentration in plasma(C_u,plasma_) (Equation (1)).

K_p,uu,brain_ = C_u,brain_/C_u,plasma_ (1)

Using microdialysis, the concentrations in brain ISF can be determined. Since the concentrations in the ISF are compared to unbound plasma concentrations to derive K_p,uu,brain_, in addition to the ISF and plasma concentrations derived from the in vivo experiment, the plasma protein binding of the investigated compound has to be determined by an in vitro method. As method development for microdialysis is time consuming and recovery can be an issue, this method is not suitable for the investigation of large numbers of compounds as typically needed in drug discovery research. A commonly used method with higher throughput is the brain homogenate method where drug concentrations in brain and plasma are determined and converted to the unbound concentrations using fraction unbound in plasma (f_u,plasma_) and fraction unbound in brain homogenate (f_u,brain_), both being determined in vitro (Equation (2)).

K_p,uu,brain_ = C_u,brain_/C_u,plasma_ = (C_brain_ × f_u,brain_)/(C_plasma_ × f_u,plasma_) = K_p,brain_ × (f_u,brain_/f_u,plasma_) (2)

where C_plasma_ and C_brain_ are the plasma and brain concentration, respectively. K_p_ is the partition coefficient of total drug in brain and plasma:

K_p,brain_ = C_brain_/C_plasma_ (3)

The assumption behind Equation (2) is that the in vitro fraction unbound in brain homogenate (f_u,brain_) equals the in vivo fraction unbound in brain. This assumption is, however, no longer valid in case of strong bases which partition strongly into acidic organelles (e.g., lysosomes). The pH partitioning is destroyed by the disruption of organelles during the preparation of brain homogenate, which leads to an overestimation of f_u,brain_ [6,7]. Measurement of volume of distribution of unbound drug (V_u,brain_) in brain slices overcomes this partitioning issue [8]; however, it is not feasible for higher throughput either. In the past, the partition coefficient of total drug, K_p_, was frequently used to describe the extent of brain distribution. Since the binding of a drug to plasma protein could differ strongly to the binding to brain tissue, use of K_p_ for this purpose can be misleading [8] and is no longer considered a state of the art approach. Instead of measuring the unbound drug concentration in ISF, drug concentration in the cerebrospinal fluid (C_CSF_) is often used as a surrogate. Because of the different physiological origin of CSF and ISF and the different expression of drug transporters in the BBB and blood–CSF barrier, the value of C_CSF_ is limited, especially for strong substrates of drug transporters [9,10,11]. Since all these methods include at least one in vivo experiment, their throughput is rather limited. For compound screening in the early drug discovery phase, several in vitro methods have been established in the pharmaceutical industry. P-gp and BCRP-transfected Madin-Darby canine kidney (MDCK) cells were shown to be valuable tools [12,13]. In such assays, the bidirectional transport of a drug across a cell monolayer grown on a Transwell^®^ insert is measured. The transport activities of P-gp or BCRP in these cells for a certain compound are expressed by the efflux ratio (ER):

ER = P_app,BA_/P_app,AB_ = 1 + J_efflux_/PS_Diff_ (4)

with P_app_AB and P_app_BA being apparent permeability coefficients in the apical to basolateral and basolateral to apical directions, respectively, J_efflux_ being the rate of transporter-mediated efflux, and PS_Diff_ the rate of passive diffusion. By setting a certain threshold for the ER, compounds can be easily filtered with regard to potential limitation in brain distribution by P-gp and/or BCRP. These in vitro data, however, have to be used with caution. Firstly, the expression levels of the transport proteins in the transfectants may differ to those in the BBB. ER measured in the in vitro assays may therefore over- or underestimate the transporter activities in vivo. Secondly, the plasma exposure of the compounds can differ strongly to the standard concentration used in the in vitro assays and over- or underestimation of transporter activities using in vitro data may occur in case of nonlinearity of the transporter activities.

In the present paper, we describe a new in vivo method for the fast estimation of in vivo K_p,uu,brain_. We validated this method with compounds from our running discovery programs by comparing it to literature known in vivo and in vitro methods. We describe the derived screening strategy applied in Boehringer-Ingelheim’s drug discovery research which helps to select compounds more efficiently and more reliably with regard to BBB penetration.

## 2. Materials and Methods

### 2.1. Compounds

Test compounds are proprietary compounds of Boehringer Ingelheim as well as reference compounds from literature and patents. They derive from a variety of different research projects. The binned distributions of molecular weight (MW), number of H acceptors (H-AC), calculated log P (clogP, Bio-Loom, BioByte Corporation, Claremont, CA, USA), total polar surface area (TPSA), and the aliphatic indicator (FSP3) are listed in Table 1.

### 2.2. Animal Studies

All animal experiments were conducted in accordance with the German and European Animal Welfare Act and authorized by the Regierungspräsidium Tübingen as the responsible local German authority under reference numbers 08-006 (21 February 2008), 11-001 (21 February 2011), and 14-009 (25 June 2014). Animal experiments were performed in male rats of different strains (Han Wistar, Sprague Dawley, Lister Hooded, Long-Evans, supplied by commercial breeders, typically Charles River, Sulzfeld, Germany or Janvier Labs, Saint-Berthevin Cedex, France). Only two studies were done in female animals. Typically three animals were used per experiment. For 10.6% of the studies which were performed in an early screening approach, only two animals were used per experiment. Compounds were dosed orally, intravenously, subcutaneously, or intraperitoneally. For tissue sampling animals were anaesthetized with Ketamin/Inactin. After dissection of the cisterna magna, approximately 50 µL CSF was collected via a 0.4-mm cannula. Blood was collected from the retroorbital plexus into K_3_EDTA coated vials (Sarstedt, Nümbrecht, Germany) and plasma was collected by centrifugation for 5 min at approximately 5000 rpm. Animals were exsanguinated via the vena cava. The femoral muscle was prepared. A small piece (approximately 0.2–0.5 g) was excised, rinsed in cold physiological NaCl, and the remaining fat and superficial vessels were removed. For brain isolation, skin and muscle of head and neck were removed. The cranium was opened with a bone trimmer by one central and two lateral incisions. Cranium and dura matter were removed and the complete brain was culled and washed in physiological NaCl. Muscle and one hemisphere of the brain were transferred into pre-weight homogenization devices (Precellys^®^ kit, 7 mL, CK28, Bertin Instruments, Montigny-le-Bretonneux, France). For homogenization, four parts (*v*/*w*) homogenization buffer (37.5% acetonitrile, 37.5% methanol, 25% water) were added. Tissues were homogenized with a Precellys^®^ Evolution homogenizer (Bertin Instruments, Montigny-le-Bretonneux, France) at 5500 rpm (three cycles of 40 s with a 30-s break in between). Five µL of the homogenate were added to 70 µL of a 1 to 1 (*v/v*) mixture of acetonitrile and methanol and frozen for at least 10 min at approximately −18 °C. For final sample preparation the sample was centrifuged at approximately 4000 rpm for 1 min. Then, 30 µL sample were mixed with 170 µL formic acid prior to injection. Calibration curve was made in plasma. In addition, six tissue quality control (QC) samples were generated. When the accuracy of the tissue QCs was within the range of ± 30% a plasma calibration curve was used, otherwise the tissue QCs were used instead of the calibration curve.

### 2.3. In Vitro Efflux Measurement

Bidirectional permeability assays were performed as described previously [14] with the exception that MDCK II cells expressing recombinant human MDR1 (MDCK-MDR1) or human BCRP (MDCK-BCRP) were used instead of Caco-2 cells. Briefly, compounds were diluted in transport buffer (128.13 mM NaCl, 5.36 mM KCl, 1 mM MgSO_4_, 1.8 mM CaCl_2_, 4.17 mM NaHCO_3_, 1.19 mM Na_2_HPO_4_, 0.41 mM NaH_2_PO_4_, 15 mM 2-[4-(2-hydroxyethyl)piperazin-1-yl]ethanesulfonic acid (HEPES), 20 mM glucose, pH 7.4) containing 0.25% bovine serum albumin to a final concentration of 1 or 10 µM and added to the apical or basolateral compartment. Cells were incubated with the compounds for up to 2 h. Samples from the opposite compartment were taken at different time points. Apparent permeability coefficients (P_app,AB_, P_app,BA_)were calculated as follows:

P_app,AB_ = Q_AB_/(C_0_ × s × t) (5)

P_app,BA_ = Q_BA_/(C_0_ × s × t) (6)

where Q is the amount of compound recovered in the receiver compartment after the incubation time t, C0 the initial compound concentration given to the donor compartment, and s the surface area of the Transwell inserts. Efflux ratio is calculated as the quotient of P_app,BA_ (mean of duplicate) to P_app,AB_ (mean of duplicate). In both MDCK-MDR1 and MDCK-BCRP assay, one reference substrate (apafant for MDR1 and daizein for BCRP) and one low permeable compound (BI internal reference, P_app_ ≈ 3 × 10^−7^ cm/s, no efflux) is included in every assay plate. In addition, Transepithelial electrical resistance (TEER) values are measured for each plate before the permeability assay. All three parameters (efflux of the reference substrates, P_app_ values of the low permeable compound, and TEER values) are used to ensure the quality of the assays.

### 2.4. In Vitro Binding Assays

Binding of research compounds to plasma protein and tissue homogenates was determined using the equilibrium dialysis method as described previously [9,15]. Briefly, plasma, brain homogenate, and muscle homogenate were spiked with 1–10 µM of test compound and dialyzed in equilibrium dialysis cells (RED-device, Thermo Scientific/Pierce or Dianorm Equlilibrium Device, Harvard Apparatus Holliston, MA, USA) against 100 mM potassium phosphate buffer pH 7.4 for 2 to 6 h at 37 °C. Unbound plasma concentration (f_u,plasma_) was calculated as:

f_u,plasma_ = (C_plasma_ − C_buffer_)/C_plasma_, (7)

where C_plasma_ and C_buffer_ are the plasma and buffer concentration, respectively. Unbound tissue concentration (f_u,tissue_) was calculated as:

f_u,tissue_ = (1/D)/(1/f_u,app_-1 + 1/D), (8)

with D being the dilution factor and f_u,app_ the observed fraction unbound in the homogenate incubations. All f_u_ values are measured in triplicate.

Compound concentrations were measured using liquid chromatography-tandem mass spectrometry (HPLC-MS/MS) according to the method described below.

### 2.5. Bioanalytics

Drug concentrations in plasma, CSF, and tissue homogenates were determined by HPLC-MS/MS (standard equipment: HPLC series 1000 or higher from Agilent, Santa Clara, CA, USA and mass spectrometers API 4000 or higher from AB Sciex, Toronto, ON, Canada). Prior to bioanalysis plasma samples and dialysates were spiked with internal standard solution and diluted with acetonitrile for protein precipitation. For CSF no protein precipitation was necessary. Measurement was operated in multiple reaction monitoring (MRM) mode. Quantification was performed using external calibration.

### 2.6. Statistical Analysis

Prediction bias was calculated as the geometric mean bias:

(9) bias=10^(∑i=1n[log(predi/obsi)]/n)

where pred_i_ and obs_i_ are the predicted and observed values of the i^th^ observation, respectively, with *n* as the total number of observations. The average fold error (AFE), which describes the average fold change of the predicted versus the observed values, was calculated as follows:

(10) AFE=10^∑i=1n[(log(predi)−log(obsi))^2]/n

Regression analysis was carried out in R [16] (Version 3.3.2). Model parameters were estimated using extended least squares [17] assuming heteroscedastic variability. The predictive performance of different models was compared with regard to the Akaike information criterion (AIC) [18], AFE, bias, and percentage of predictions falling within the two- and three-fold error range.

## 3. Results

### 3.1. K_p,brain/muscle_ (K_p,br/mu_) as a Surrogate for In Vivo Blood–Brain Barrier Efflux

Analogous to Equation (2), the K_p,uu_ of any tissue can be described as

K_p,uu,tissue_ = C_u,tissue_/C_u,plasma_ = (C_tissue_ × f_u,tissue_)/(C_plasma_ × f_u,plasma_) = K_p,tissue_ × (f_u,tissue_/f_u,plasma_) (11)

In a tissue without substantial drug transporter mediated active uptake or efflux, K_p,uu_ approaches 1 under steady-state conditions. Equation (9) can be transformed to

K_p,tissue_ = f_u,plasma_/f_u,tissue_ (12)

By combining Equations (2) and (12), the ratio between the partition coefficient of brain and this tissue can be expressed as

K_p,brain_/K_p,tissue_ = (f_u,plasma_ × f_u,tissue_ × K_p,uu,brain_)/(f_u,brain_ × f_u,plasma_) = K_p,uu,brain_ × (f_u,tissue_/f_u,brain_) (13)

with rearrangement leading to

K_p,uu,brain_ = (K_p,brain_/K_p,tissue_) × (f_u,brain_/f_u,tissue_) = (C_brain_/C_tissue_)/(f_u,brain_/f_u,tissue_) (14)

As suggested by Equation (16), the K_p,uu,brain_ of a compound could be simply determined without in vitro measurement of f_u_ in plasma or tissue homogenate as the ratio of the exposure in brain and in a tissue without efflux and with a similar free fraction as in brain:

K_p,uu,brain_ ≈ C_brain_/C_tissue_ = K_p,brain/tissue_, if f_u,brain_≈ f_u,tissue_ (15)

Published data show that skeletal muscle has similar fractions of neutral lipids, neutral phospholipids, acidic phospholipids, and intracellular and extracellular tissue water, as well as similar tissue-to-plasma ratios of albumin and lipoprotein as compared to the brain [19,20]. Tissue binding in brain and muscle could be expected to be very similar. Indeed, compounds without blood–brain barrier efflux like midazolam or diazepam show very similar distributions into brain and skeletal muscle [19,20]. Our in-house next generation sequencing (NGS) data indicate very low levels of expression of drug transporters like P-gp and BCRP in skeletal muscle (data not shown). All these data suggest that skeletal muscle could be used as an in vivo reference tissue for a rapid estimation of K_p,uu,brain_ as described in equation (17).

**Figure 1:**
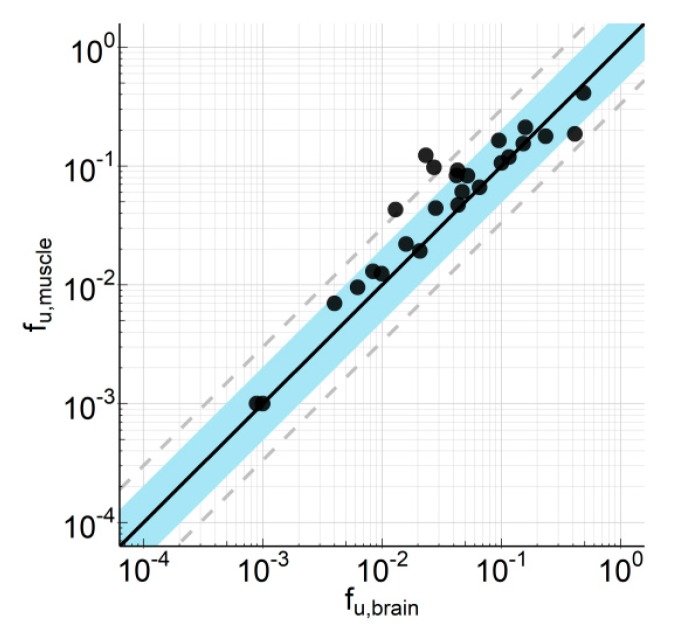
Correlation of fraction unbound in brain homogenate (f_u,brain_) and muscle homogenate (f_u,muscle_). Unbound brain and muscle concentrations were determined by means of equilibrium dialysis of compound spiked into tissue homogenate. Experiments were performed in triplicate. The compounds derive from 10 different research projects with a molecular weight (MW) range from 305 to 563, a clogP range from 0.7 to 5.2, a total polar surface area (TPSA) from 46 to 119, an H-acceptor range from 2 to 8, and a FSP3 range from 0.19 to 0.62. Data are listed in Supplement Table S1.

As a validation of this hypothesis, we measured the f_u_ of 25 internal compounds in muscle and brain homogenate of rat. As depicted in Figure 1, there is a good correlation between the f_u_ in these two tissues with an average fold error (AFE) of 1.82, a bias of 0.721, and 80.8% and 88.5% of compounds within 2-fold and 3-fold error, respectively.

Based on these data and Equation (15), K_p,uu,brain_ can now be described as follows (K_p,brain/muscle_ will be abbreviated as K_p,br/mu_ in the remaining text):

K_p,uu,brain_ ≈ C_brain_/C_muscle_ = K_p,brain/muscle_ (16)

### 3.2. Comparison of K_p,Br/Mu_ With K_p,Uu,Brain_ Derived from the Brain Homogenate Method and CSF

**Figure 2:**
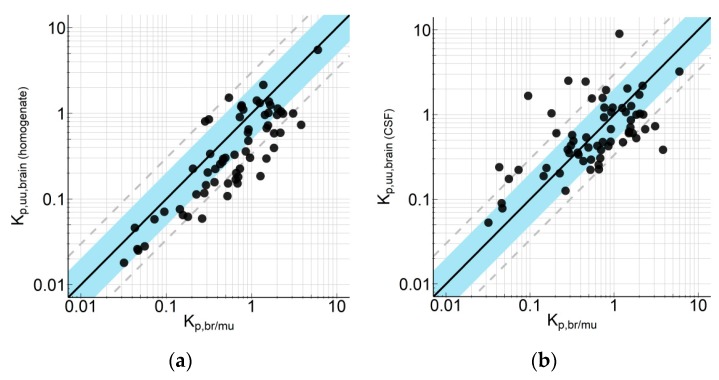
Correlation of the partition coefficient of a drug between brain and skeletal muscle (K_p,br/mu_) with (**a**) partition coefficient of unbound drug (K_p,uu_) calculation using the brain homogenate method for f_u,brain_ estimation (K_p,uu,brain(hom)_) and (**b**) C_CSF_ as a surrogate for C_u,brain_ (K_p,uu,CSF_). Symbols represent average values (*n* = 2–3). The compounds derive from 10 different research projects with a MW range from 194 to 589, a clogP range from 1.0 to 5.2, a TPSA from 25 to 122, a number of H-acceptor range from 1 to 7 and a FSP3 range from 0.07 to 0.62. Data are listed in Supplement Table S2.

In order to further validate Equation (16), we compared the K_p,br/mu_ of 64 internal compounds with two methods frequently used for the determination of BBB efflux: K_p,uu_ calculation using the brain homogenate method for f_u,brain_ estimation (K_p,uu,brain(hom)_) and the method using C_CSF_ as a surrogate for C_u,brain_ (K_p,uu,CSF_). The correlations are depicted in Figure 2. The statistical analysis indicated a good correlation between K_p,br/mu_ and K_p,uu,brain(hom)_, with an AFE of 2.42, a bias of 1.80, and 53.1% and 75.0% of data within a 2-fold and 3-fold error, respectively. The correlation between K_p,br/mu_ and K_p,uu,CSF_ as surrogate for K_p,uu,brain_ was comparable with an AFE of 2.59, a bias of 0.998, and 56.3% and 81.3% of data within a 2-fold and 3-fold error, respectively. Interestingly, a simple regression analysis indicates that the correlation between K_p,br/mu_ and K_p,uu,brain(hom)_ (R^2^ 0.721) was clearly better than between K_p,br/mu_ and K_p,uu,CSF_ (R^2^ 0.365).

### 3.3. Robustness and Versatility of the K_p,Br/Mu_ Method

As a further characterization, we checked also the robustness of our new method with one compound being repeatedly tested in independent experiments. The variability of K_p,br/mu_ values from all experiments was less than 2-fold (Supplement Figure S1).

Typically, the tissues were sampled around the time at maximum plasma compound concentration (T_max_) after oral administration. For selected compounds, we investigated the time-dependence of K_p,br/mu_. As shown in Table 2, the influence of sampling time was negligible.

**Figure 3:**
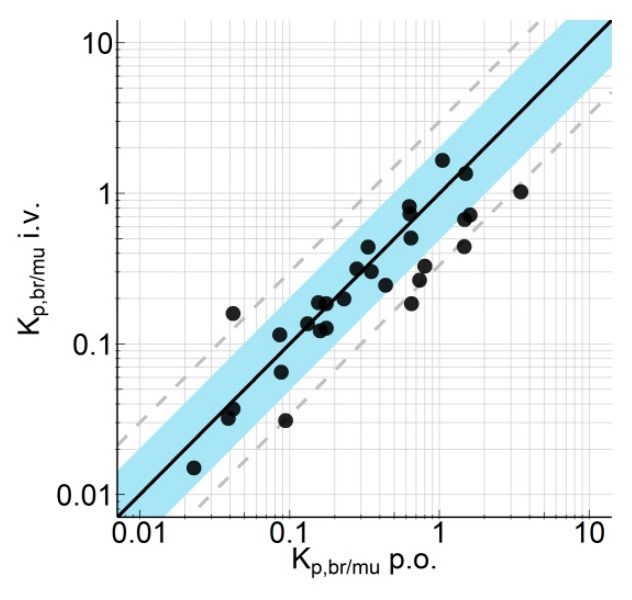
Correlation of K_p,br/mu_ values obtained following intravenous and oral dosing for a set of 29 compounds. Symbols represent average values (*n* = 2–3). Data are listed in Supplement Table S3.

Also, the application route did not influence the K_p,br/mu_ of a certain compound. The correlation of K_p,br/mu_ determined after oral and intravenous administration of 29 compounds is depicted in Figure 3. It was found that −67.0% and 82.8% of data were within 2-fold and 3-fold deviation, respectively. Together with the calculated AFE of 1.91 and bias of 0.757 this indicates a good correlation. The same was true for subcutaneous and intraperitoneal application (data not shown).

### 3.4. Correlations Between K_p,Br/Mu_ and In Vitro Efflux Measured with Transfected Cells

**Figure 4:**
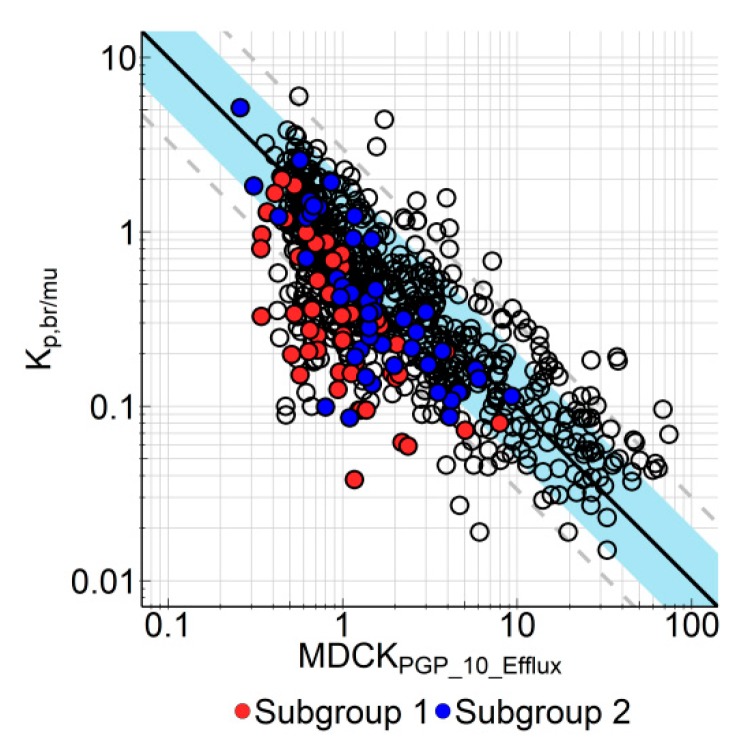
In vitro–in vivo correlation of efflux in multidrug resistance 1 transfected Madin-Darby canine kidney (MDCK-MDR1) cells and K_p,br/mu_. K_p,br/mu_ is depicted as function of efflux derived from in vitro transporter experiments at 10 µM compound concentration in MDCK-MDR1 cells (MDCK_Pgp_10_Efflux_). Regression analysis was performed with all data depicted in this plot. Solid line represents regression, blue area indicates the 2-fold error range, dashed lines are 3-fold error lines. Compounds from subgroup 1 (red circles) and subgroup 2 are highlighted because they were tested in additional in vitro transporter assays (see Figure 5; Figure 6 and corresponding text). Symbols represent average values (*n* = 2–3 for K_p,br/mu_ and *n* = 2 for in vitro efflux). Data are listed in Supplement Table S4.

Bidirectional permeability assay using MDR1-transfected MDCK cells is a well-accepted in vitro screening assay in the pharmaceutical industry to address the blood–brain barrier efflux [12,13,21]. However, since the expression levels of the transporters in the cell lines used in different labs may differ strongly [21], it is important for each lab to calibrate its in vitro screening assay using in vivo data. In our case we compared the in vitro efflux measured in our in-house MDR1-overexpressing MDCK cells (MDCK-MDR1) with K_p,br/mu_ measured in rats. Figure 4 shows the correlation between Efflux measured in MDCK-MDR1 cells with a substrate concentration of 10 µM and K_p,br/mu_ for 681 BI research compounds. As some of these compounds were dosed by more than one administration route, the dataset consists of 713 data points. In general, there is a good correlation between the in vitro and in vivo data (AFE 2.34, bias 0.732, 64.6% and 83.1% of data within 2-fold and 3-fold error, respectively).

**Figure 5:**
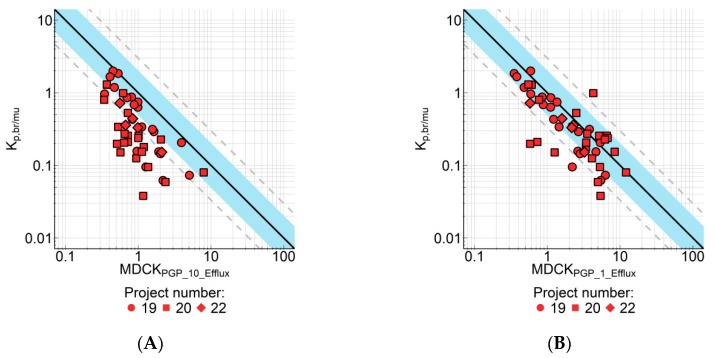
Correlation of K_p,br/mu_ with in vitro efflux measured in MDCK-MDR1 cells for compounds of three research projects (*N* = 48). The in vitro assay was performed at compound concentration of 10 µM (**A**) or 1 µM (**B**). Form of the symbols indicates individual research projects. Solid line represents regression; blue area is the 2-fold error range, dashed lines are 3-fold error lines. Symbols represent average values (*n* = 2–3 for K_p,br/mu_ and *n* = 2 for in vitro efflux). Data are listed in Supplement Table S5.

However, as is obvious in Figure 4, some compounds did show a disconnect between in vitro and in vivo efflux. These are highly permeable compounds with no or low in vitro P-gp efflux; however in tissue distribution studies the K_p,br/mu_ of these compounds indicated higher in vivo efflux than expected from the in vitro data. After in-depth profiling of these compounds, we could divide these compounds into two subgroups. For one subgroup, the explanation for this disconnect is the rather high concentration (10 µM) in the MDCK-MDR1 assay. Due to the non-linear nature of P-gp transporter activities, the ratio between the rate of efflux and the rate of passive diffusion may decrease with increasing substrate concentration. In such cases, reducing substrate concentrations could improve the in vitro–in vivo correlation (IVIVC) since the plasma exposures in the animal studies were usually much lower than 10 µM. Indeed, when measured at 1 µM in the MDCK-MDR1 assay, the IVIVC for this subgroup of compounds was improved (Figure 5). AFE decreased from 4.00 to 2.00, bias increased from 0.301 to 0.647, and number of compounds within 2-fold/3-fold error increased from 25.0%/47.9% to 70.8%/85.4%, respectively.

**Figure 6:**
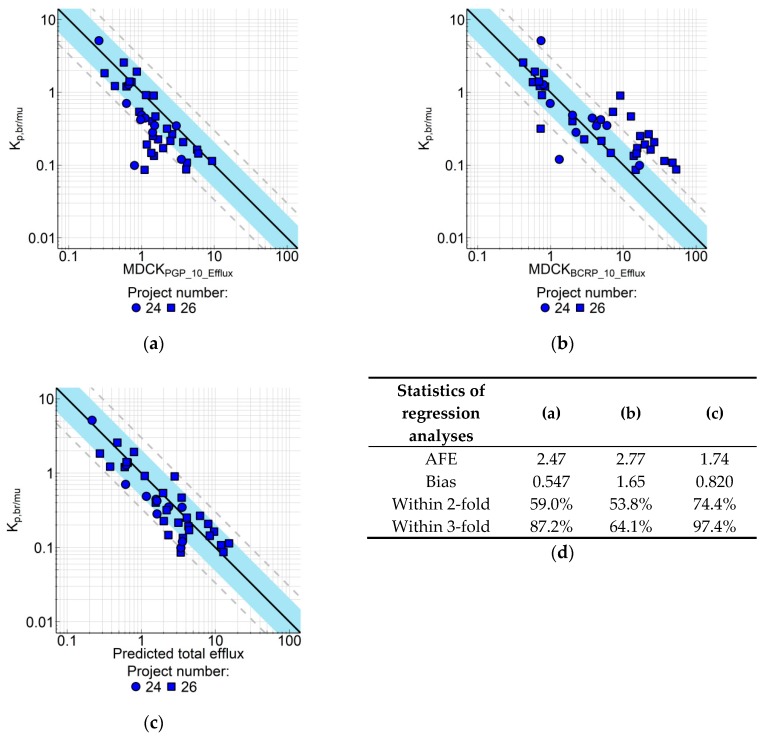
Correlation of K_p,br/mu_ with in vitro efflux measured in MDCK-MDR1 and breast cancer resistance protein transfected Madin-Darby canine kidney (MDCK-BCRP) cells for compounds from two research projects (*n* = 39). In vitro efflux determined at 10 µM compound concentration in (**a**) MDCK-MDR1 cells or (**b**) MDCK-BCRP cells was correlated with K_p,br/mu_. (**c**) Correlation of K_p,br/mu_ with in vitro efflux calculated using equation (17). (**d**) Regression line statistics. Solid line represents regression; blue area is the 2-fold error range, dashed lines indicate 3-fold error. Symbols represent average values (*n* = 2–3 for K_p,br/mu_ and *n* = 2 for in vitro efflux). Data are listed in Supplement Table S6.

For the second subgroup of compounds with IVIVC disconnect, the explanation is the involvement of BCRP, another major drug transporter at the BBB. When tested in the MDCK-BCRP assay, most of these compounds turned out to be BCRP substrates, and some are substrates for both P-gp and BCRP. When correlated with K_p,br/mu_, the in vitro BCRP efflux strongly overestimated the in vivo efflux (Figure 6B). The overestimation was mainly due to the very high expression of the recombinant BCRP in our cells (in-house proteomic data). In order to correct the in vitro efflux by the expression level, we used a similar approach as described by Liu et al. [13]: For the BCRP-selective substrate dantrolene [9,13,22] we determined both the in vitro efflux (40.3) in MDCK-BCRP cells and the K_p,br/mu_ (6.2) in rats, which resulted in a correction factor of about 6. Assuming that the total efflux at the BBB is the sum of efflux by both transporters, the total in vitro efflux from P-gp and BCRP can be calculated as follows:

Efflux_total_ = (Efflux_Pgp_ − 1) + ((Efflux_BCRP_ − 1)/6) + 1 (17)

As shown in Figure 6C, the IVIVC is strongly improved for this subgroup of compounds by using the total in vitro efflux.

Instead of using the correction factor determined with the reference BCRP substrate dantrolene, we tried also to determine correction factors for both in vitro assays with an unbiased approach. As described in Equation (18),

Efflux_total_ = ((Efflux_Pgp_ − 1)/Θ_PGP_) + ((Efflux_BCRP_ − 1)/Θ_BCRP_) + 1 (18)

Regression models including correction factors Θ_PGP_ for the term (Efflux_Pgp_ − 1) and Θ_BCRP_ for (Efflux_BCRP_ − 1) were fitted to data from 133 compounds. The results are listed in Table 3. The model where Θ_BCRP_ was estimated and Θ_PGP_ was fixed to 1 (BCRP model) resulted in a similar value for this parameter compared to the fixed model as described by Equation (17). While both models were comparable in terms of AIC and AFE, the BCRP model was slightly less biased (calculated bias closer to 1) than the fixed model, but fewer predictions fell within the two- and three-fold error range. Overall, both models performed equally well, confirming the validity of determining the BCRP-related efflux correction directly from the dantrolene in vitro data. Estimating the correction factors for both efflux terms resulted in the numerically best fit, as shown by the lowest AIC and the lowest AFE of all tested models. The difference to the fixed and the BCRP model was, however, only marginal. Sole estimation of Θ_PGP_ (P-gp model), which lacks a scientific rationale, was inferior to all other methods.

## 4. Discussion

We describe in this publication a new method for the rapid estimation of the in vivo BBB efflux of a compound by using the partition coefficient K_p,br/mu_. The underlying assumptions are: (1) that binding within brain and muscle tissue, which is based upon pH partitioning and binding to neutral lipids, neutral phospoholipids and acidic phospholipids [19,20] is comparable due to similar intracellular pH and similar amounts of the different phospholipids in both tissues; and (2) that the efflux transporters which limit distribution into brain, especially P-gp and BCRP, are not expressed in muscle tissue in relevant amounts.

Since the pH of the intracellular water of both brain and muscle tissue is reported to be identical, pH 7.0 [19], pH partitioning should not contribute to differences in distribution of drugs between both tissues. The fractional tissue volume of neutral lipids is approximately four-fold higher in brain than in muscle tissue, whereas the tissue concentration of acidic phospholipids is approximately fourfold higher in muscle than in brain tissue [19,20]. According to the models of Rodgers and Rowland, the dominating factor for moderate to strong bases is binding to acidic phospholipids, whereas for weak bases, acids, and neutral compounds it is binding to neutral lipids [19,20]. Calculation of the distribution of unbound drug between plasma and both tissues (K_p,u,brain_ and K_p,u,muscle_) for the compounds described in both publications shows that both values typically are within a threefold range, what leads to comparable values for the tissue partitioning coefficients K_p,u,brain_ and K_p,u,muscle_. Consistent with these considerations, we could show for 26 compounds (for which calculated or measured pKa values ranged between 2 and 11) very similar binding in homogenates from brain and skeletal muscle of rats (Figure 1). Assuming that transporters do not relevantly affect the distribution into muscle, differences in the tissue partitioning coefficients can therefore be attributed to transporters at the blood–brain barrier. That allows us to estimate efflux at the blood–brain barrier (K_p,uu,br_) using the ratio of the tissue partitioning coefficients K_p,u,brain_/K_p,u,muscle_ and with the further assumption of identical binding in tissues (f_u,brain_ = f_u,muscle_) to use the ratio K_p,br/mu_. However, for weak bases, acids, and neutral compounds, K_p,u,brain_ is typically slightly greater than K_p,u,muscle_ and for moderate to strong bases K_p,u,muscle_ is typically slightly greater than K_p,u,brain_, what would lead to a small overestimation of free brain concentration and underestimation of efflux for weak bases, acids and neutral compounds and a small underestimation of free brain concentration and overestimation of efflux for moderate to strong bases (data not shown). It should be mentioned that we have not done a systematic comparison of partitioning within a broad set of different tissues. Although we see muscle as an appropriate reference tissue, there is a possibility that other tissues could also be suitable or even better for this purpose.

Typical drug transporters like organic anion transporting polypetide (OATP) 1B1, OATP1B3, organic anion transporter (OAT) 1, OAT3, P-gp, BCRP, and multidrug resistance protein (MRP) 2 are not detected in skeletal muscle [23]. However, OATP2B1, MRP1, MRP4, and MRP5, which are relevant for the uptake (OATP2B1) and efflux (MRPs) of statins (e.g., atorvastatin and rosuvastatin) in skeletal muscle, were detected in skeletal muscle by mRNA expression, western blot, and immunolocalization [23]. Thus, if other transporters than P-gp or BCRP are involved in the brain and/or muscle distribution of a drug the potential role of these transporters has therefore to be carefully assessed before applying this method. On the other hand, discrepancies between in vitro efflux data for P-gp/BCRP and K_p,uu,brain_ determined using K_p,br/m_ or between K_p,uu,brain_ determined using K_p,br/m_ and other methods can be an indication that other transporters or mechanisms are potentially involved.

Our results demonstrate that the new method described here is fast, robust, and versatile and provides sufficient comparability to the standard method for the estimation of BBB efflux (K_p,uu,brain_), the brain homogenate method (K_p,uu,brain(hom)_). It is certainly debatable whether the K_p,uu,brain_ calculation using f_u,brain_ determined from brain homogenate is appropriate for all compounds, e.g., the unbound fraction of basic compounds with strong tendency of pH partitioning could be overestimated [6,7,8]. In this regard, the K_p,br/mu_ method could be even superior to the K_p,uu,brain_ calculation using f_u,brain_ determined from brain homogenate method since the pH partitioning into brain and skeletal muscle seems to be similar [24]. A more accurate method to determine the unbound concentration of basic compounds in brain is the determination of V_u,brain_ with the brain slice method [6,7,8]. Due to the limited throughput of this method, however, it is not suitable for large-scale compound screening. Moreover, most of our compounds with CNS targets are neutral or weakly basic. For those compounds, the effect of pH partitioning on tissue distribution is considered minor. Indeed, we measured the V_u,brain_ of a small set of our compounds and could confirm the minor effect of pH partitioning on the K_p,uu,brain_ calculation using the brain homogenate method (data not shown). For the brain homogenate method three separate experiments are necessary: one in vivo tissue distribution study and two in vitro studies to determine f_u,plasma_ and f_u,brain_. For K_p,br/mu_ determination, one single in vivo tissue distribution study is sufficient. In addition to the lower experimental effort needed, K_p,br/mu_ determination is considered less prone to experimental variability than the brain homogenate method: Since experimental variability is intrinsic in all data, the more data points are used in a calculation, the stronger the amplification of the variability. Especially for highly bound compounds, the unbound fraction measurement in the screening assays is often hampered by the limit of quantification in the analytical assay, despite the use of LC-MS/MS. In those cases the K_p,uu,brain_ calculation using the brain homogenate method can become unreliable. Here, K_p,br/mu_ measurement provides a more reliable estimation of in vivo efflux at BBB as it does not need those very high sensitivity of the analytical methods. As discussed earlier [9,11], CSF is a less reliable surrogate for C_u,brain_ compared to other methods when the compounds are substrates of efflux transporters. The poorer correlation between K_p,uu,CSF_ and K_p,br/mu_ compared to K_p,uu,brain_ and K_p,br/mu_ in our experiments (Figure 2) confirm this.

A few compounds tested are reference compounds with published brain partitioning data. As shown in Table 4, K_p,uu,brain_ values of the P-gp substrate quinidine and the non-efflux compound carbamazapine reported by Kodaira et al. fit well to our K_p,br/mu_ values [11]. For the BCRP substrate dantrolene, however, there is a strong discrepancy between the value reported by Kodaira et al. and the K_p,br/mu_ in this study. Interestingly, our K_p,uu,brain_ value determined using the brain homogenate method is only 2-fold higher than that reported by Kodaira et al., suggesting that the discrepancy might not result from lab-to-lab variability, but rather from the difference in the methods. Moreover, brain distribution studies with dantrolene in Bcrp knockout rats demonstrated that dantrolene is rather a low efflux substrate with a K_p,uu,brain_ of 0.27 [22], very close to our K_p,br/mu_ value. Since K_p,uu,brain_ calculation using brain slice method or brain homogenate method includes an in vitro f_u,brain_ determination, whereas distribution studies using knockout animals and the K_p,br/mu_ method include only in vivo measurements, the discrepancy might represent a underestimation of f_u,brain_ by the in vitro measurements. Consistent to this consideration, we observed a 2-fold bias of K_p,br/mu_ towards higher values compared to K_p,uu,brain_ values (Figure 2A). Underestimation of K_p,uu,brain_ might result from the underestimation of f_u,brain_ by the in vitro method.

Bidirectional permeability measurement using transfected MDCK cells is a well-accepted in vitro screening assay in the pharmaceutical industry to address the blood–brain barrier efflux [12,13,21]. We describe in this work the calibration of our in-house transfected cell lines with the in vivo K_p,br/mu_ data. The IVIVC is in general very good with regard to P-gp mediated efflux, although the substrate concentration in the permeability assay has to be reduced for certain compound classes. Our standard substrate concentration of 10 µM was a balance between assay efficiency and relevance of the results. The unbound plasma concentration of a test compound in a pharmacokinetic (PK) or a pharmacodynamics (PD) study is in most cases lower than 10 µM, therefore a lower substrate concentration in the in vitro assay would be more appropriate. However, especially for compounds with lower permeability or those with bad ionization behavior, sensitivity issues of the LC-MS/MS methods prevent investigations at lower substrate concentration. A retrospective analysis showed that for ~20% of our research compounds the limit of quantification would be insufficient for a substrate concentration of 1 µM (data not shown). Using such a concentration as standard substrate concentration would lead to too many repeats of the experiments at higher concentrations. Our screening strategy was thus to get an IVIVC using standard substrate concentration of 10 µM at an early stage of a research project. In case of insufficient correlation, the substrate concentration can be reduced to 1 µM accompanied by investment in improving the analytical methods for these specific compounds/compound classes. Otherwise, IVIVC needs to be spot-checked regularly.

For some of compounds, insufficient IVIVC with MDCK-MDR1 cells was not resolved by applying a lower substrate concentration, but rather caused by the involvement of other transporters at the BBB. The MDCK-BCRP assay was a valuable tool to identify such compounds. Due to the very high expression level of recombinant BCRP in the transfected cells, a correction factor has to be applied for translation of the in vitro efflux to in vivo efflux. Using the reference BCRP substrate dantrolene, we determined a correction factor of about 6. A similar approach was already described by Liu et al. [13]. Since some compounds are substrates of both P-gp and BCRP, estimation of in vivo efflux needs to combine data from both in vitro assays. Although a synergism between P-gp and BCRP at the BBB was hypothesized [25], the total efflux of both transporters is considered rather additive [26]. Based on these considerations, we developed Equation (17) to calculate a total in vitro efflux using data from both in vitro assays. The validity of this equation was confirmed by the regression analysis of data from a set of 133 compounds (Table 3). As the expression of recombinant transporters differs from laboratory to laboratory, our equation is essentially laboratory-specific and needs an individual calibration for the respective transfected cell lines in the different laboratories. It is important to note that all in vitro data were obtained with cells expressing recombinant human transporters and the in vivo data were from rats. The reported similar activities of rodent and human P-gp in vitro is consistent with the good IVIVC we observed here [12]. Quantitative proteomics indicates a three to four-fold higher P-gp protein concentration in the BBB of rat than in human BBB, while BCRP protein concentrations are comparable in rats and humans [27,28]. Consistent with these data, positron emission tomography studies with [^11^C]verapamil indicated a roughly three-fold higher BBB efflux of Verapamil in rat than in human [29]. However, Braun et al. demonstrated in dogs that up to 5-fold expression differences of P-gp as well as BCRP between cerebrum, cerebellum, and brain stem of dogs did not affect the K_p,brain_ of quinidine, apafant, dantrolene, and daidzeine, two P-gp and two BCRP substrates in these brain regions of the same dogs [9]. Thus it is currently unclear whether differences in transporter expression really affect the drug concentrations in brain. Due to the proposed, at maximum three fold difference between rats and humans, it is thus reasonable to assume that the efflux measured in rats is close to that in humans. The good correlation between in vivo data in rat and data from our in vitro assays therefore qualifies these assays for compound screening and selection of development candidates.

The rat is our primary species for pharmacokinetic optimization and thus also used in screening for optimization of BBB penetration since the available plasma pharmacokinetic profiles allow to select appropriate doses and time points for the brain distribution experiments. Since the mouse is often used for pharmacology studies there is also an interest in assessing the BBB efflux in this species. For a set of 135 compounds we investigated the correlation between in vitro efflux derived from MDCK-MDR1 cells with the mouse K_p,br/mu_. We found, as for the rat, there was a good correlation, as depicted in Supplement Figure S2 (data in Table S7). Thus, the method is in principle also working in mice, which can be rationalized by the comparable expression of P-gp and BCRP in the BBB of rat and mouse (BCRP mouse/rat: 4.41 ± 0.69 fmol/µg protein/4.95 ± 0.32 fmol/µg protein; P-gp mouse/rat: 14.1 ± 2.1 fmol/µg protein/19.1 ± 1.0 fmol/µg protein) [27,28].

**Scheme 1:**
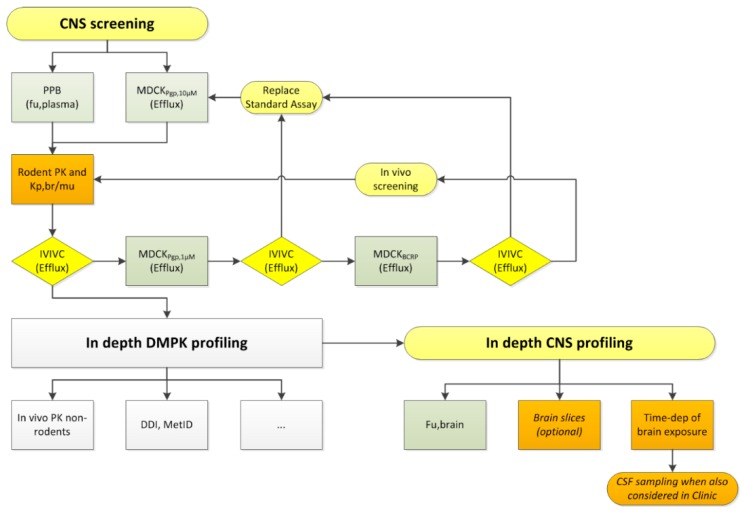
Screening cascade with focus on the central nervous system (CNS) specific ADME (absorption, distribution, metabolism, excretion) assays. “…” = placeholder for additional profiling assays. CSF = cerebrospinal fluid; DDI = drug-drug interaction; DMPK = drug metabolism and pharmacokinetics; IVIVC = in vitro–in vivo correlation; MetID = metabolite identification; PK = pharmacokinetics; PPB = plasma protein binding; Time-dep = time dependence;

Our CNS screening strategies have changed over the last decade(s), driven by better understanding of the BBB as well as new assays being available for screening and in depth investigations. While considering CSF as major determinant of unbound brain concentrations in the past, we moved to determination of K_p,uu,br_ and now K_p,br/mu_ as efficient in vivo approach. Even more influence on our screening cascades came from the availability of the efflux transporter expressing MDCK cells. The improved predictivity of the in vitro assays generated the necessary trust in this data, thus ultimately reduced the number of compounds tested in vivo and sped up the decision process. Scheme 1 depicts the CNS-specific ADME (absorption, distribution, metabolism, excretion) assays of a generic screening cascade applied at Boehringer Ingelheim. It applies to compounds with a reasonable potency, selectivity, and metabolic stability. In the CNS screening part of the cascade, in vitro efflux and f_u,plasma_ are measured in tier one. Usually, the MDCK-MDR1 assay is used as first line screening assay. As discussed above, spot-checking of the IVIVC by pharmacokinetic (PK) studies with the measurement of K_p,br/mu_ is performed in order to determine the optimal substrate concentration in the MDCK-MDR1 assay or the necessity of including the MDCK-BCRP assay. During regular compound screening, Equation (17) is used to calculate the total in vitro efflux.

There are two ways to rank the compounds with regard to efflux. If high systemic exposure is undesired, e.g., due to target-related side effects in peripheral tissues, a general cutoff can be used to filter out high-efflux compounds. Taking all experimental variabilities into account, a K_p,br/mu_ greater than 0.3 indicates a rather low or negligible efflux at BBB. A lower cutoff is conceivable depending on the nature of the off-targets and the associated side effects. If the question is whether or not a compound could achieve efficacious exposure in brain with a realistic plasma exposure at all, in vitro potency, in vitro efflux, and f_u,plasma_ can be used in combination to estimate the total plasma exposure (C_plasma,total_) to be achieved in a pharmacodynamic (PD) study based on a simple equation:

C_plasma,total_ = (potency_in vitro_ × efflux_in vitro_)/(f_u,plasma_) (19)

Together with the in vitro data of metabolic stability, compounds can be rank-ordered for further profiling. Both approaches helped to reduce the number of compounds to be tested in PK and/or PD studies. In 2007, when the MDCK assay had not yet been established, brain exposure of nearly 500 compounds was investigated in rats through routine CSF sampling. In 2018, after implementation of our strategy, around 2000 Boehringer Ingelheim compounds were tested in at least one of the two in vitro efflux assays. Only about 100 compounds were further tested in a PK study with the measurement of K_p,br/mu_. For development candidates, we perform in depth CNS profiling to further improve understanding of the compound(s). This includes determining K_p,uu,brain_ with the brain homogenate method and investigation of the time-dependence of brain exposure. Microdialysis and measurement of V_u,brain_ in brain slices (both considered to generate the most accurate data for estimation of unbound brain concentration) and in vivo studies with efflux transporter knock out animals are considered when we observe discrepancies in the available BBB data or an exact (less than 2-fold error) prediction of the human free brain concentrations is essential for the project. In case CSF sampling is considered for clinical development, we investigate this also in animals to provide the necessary translational data. Besides the CNS profiling, a battery of candidate profiling studies are done, such as in vitro drug-drug interaction (DDI) assessment and in vitro as well as in vivo metabolite identification (MetID) studies, via non-rodent PK studies enabling human PK prediction to perform selection and general pharmacology investigations.

## 5. Conclusions

In summary, we describe here the establishment and validation of a new method to use the partition coefficient K_p,br/mu_ as a descriptor of in vivo efflux at the BBB. K_p,br/mu_ in rats correlates well with the more broadly used K_p,uu,brain_ using the brain homogenate method and the in vitro efflux measured in our in-house transfected MDCK cell lines. The combination of both in vitro and in vivo tools ensures a fast and efficient compound screening and optimization procedure during drug discovery. Despite the good comparability between K_p,br/mu_ and K_p,uu,brain_, using the brain homogenate method we are aware of the fact that K_p,br/mu_ is only an approximation of the real in vivo efflux at the BBB. Nevertheless, it is a helpful parameter for speeding up compound screening and for shortening optimization cycles. For compounds approaching candidate selection, we measure f_u,brain_ or, if necessary, V_u,brain_, and use K_p,uu,brain_ for the estimation of in vivo efflux.

## Figures and Tables

**Table 1:** Distribution of binned (Bin) molecular descriptors of the investigated compounds.

Bin	MW^1^	H-AC^2^	ClogP^3^	TPSA^4^	FSP3^5^
1	2	4	9	2	8
2	4	19	45	10	52
3	33	68	95	29	205
4	115	166	158	47	187
5	158	226	230	88	137
6	174	169	119	103	64
7	130	22	17	159	17
8	46	2	3	94	6
9	12	--	--	88	--
10	2	--	--	39	--
11	--	--	--	13	--
12	--	--	--	4	--

^1^ Molecular weight (MW): from ≤200 (Bin 1) to >600 (Bin 10) with increments of 50. ^2^ Number of H acceptors (H-AC): from 1 (Bin 1) to 8 (Bin 8) with increments of 1. ^3^ Calculated log P (clogP): from ≤0 (Bin 1) to >6 (Bin 8) with increments of 1. ^4^ Total polar surface area (TPSA): from ≤30 (Bin 1) to >130 (Bin 12) with increments of 10. ^5^ Aliphatic indicator (FSP3): from ≤0.1 (Bin 1) to >0.7 (Bin 8) with increments of 0.1. --: empty bin.

**Table 2:** Time dependency of K_p,br/mu_ values for five compounds (Cpd). Tissues were collected at the indicated time points after oral administration of the compounds. Data are mean ± SD, *n* = 3.

Time (h)	Cpd 5	Cpd 168	Cpd 270	Cpd 603	Cpd 609
0.5	0.089 ± 0.011			0.339 ± 0.032	2.13 ± 0.07
1			0.039 ± 0.002		
2	0.144 ± 0.087	0.666 ± 0.153			2.37 ± 1.52
4				0.348 ± 0.082	
6			0.089 ± 0.005		
8		0.664 ± 0.086			
9			0.080 ± 0.013		
24		0.544 ± 0.072			

**Table 3:** Regression analysis for K_p,br/mu_ and efflux models. For the fixed model, the correction factor for the term Efflux_Pgp_ − 1 (Θ_PGP_) and the correction factor for the term Efflux_BCRP_ − 1 (Θ_BCRP_) in Equation (18) were set to 1 and 6, respectively. For the P-gp model, Θ_PGP_ was estimated and for the BCRP model Θ_BCRP_ was estimated. For the full model, correction factors for both terms were estimated. AIC = Akaike information criterion, AFE = average fold error, Bias = geometric mean bias, RSE = relative standard error of the correction factor estimate.

Parameter	Fixed Model	P-gp Model	BCRP Model	Full Model
AIC	606	666	605	600
Θ_BCRP_	6 (fixed)	1 (fixed)	4.28	3.43
Θ_PGP_	1 (fixed)	2.18	1 (fixed)	1.24
AFE	2.06	2.46	2.06	2.02
Bias	0.850	0.948	0.879	0.876
Within 2-fold	71.4%	53.4%	67.7%	69.9%
Within 3-fold	89.5%	78.9%	88.7%	89.5%

**Table 4:** Comparison of brain partitioning data in rats from this study with literature data.

Drug	K_p,uu,brain*_ (Literature)	K_p,uu,brain(hom)**_ (This publication)	K_p,br/mu_ (This publication)
Carbamazapine	0.771		1.44
Dantrolene	0.0297	0.061	0.19
Quinidine	0.026	0.0276	0.056

*: [11], Partition coefficient of unbound drug between brain and plasma (K_p,uu,brain_) determined with brain slice method.; **: K_p,uu,brain_ determined with brain homogenate method.

## Supplementary Materials

The following are available online at https://www.mdpi.com/1999-4923/11/11/595/s1. Table S1: Raw data Figure 1 (Correlation of f_u,brain_ and f_u,muscle_.); Table S2: Raw data Figure 2 (Correlation of K_p,br/mu_ with (a) K_p,uu,brain(hom)_ and (b) K_p,uu,CSF_.); Table S3: Raw data Figure 3 (Correlation of K_p,br/mu_ values obtained following intravenous and oral dosing for a set of 29 compounds.); Table S4: Raw data Figure 4 (In vitro–in vivo correlation of MDR1 P-gp efflux and K_p,br/mu_.); Table S5: Raw data Figure 5 (Correlation of K_p,br/mu_ with in vitro efflux measured in MDCK-MDR1 cells for compounds of three research projects (*n* = 48)).; Table S6: Raw data Figure 6 (Correlation of K_p,br/mu_ with in vitro efflux measured in MDCK-MDR1 and MDCK-BCRP cells for compounds from two research projects (*n* = 39)). Table S7: Raw data Figure S2 (In vitro–in vivo correlation of MDCK P-gp efflux and mouse K_p,br/mu_ (*n* = 135)); Figure S1: Reproducibility of K_p,br/mu_; Figure S2: In vitro–in vivo correlation of MDR1 P-gp efflux and mouse K_p,br/mu_.
