# Supplementary material for: Muscle to Brain Partitioning as Measure of Transporter-Mediated Efflux at the Rat Blood–Brain Barrier and Its Implementation into Compound Optimization in Drug Discovery
Source: Pharmaceutics. 2019 Nov 11;11(11):595. doi: 10.3390/pharmaceutics11110595 (PMC6920949; doi:10.3390/pharmaceutics11110595)
Supplement: Supplementary file 1 [file pharmaceutics-11-00595-s001.pdf]

pharmaceutics

MDPI

Muscle to Brain Partitioning as Measure of Transporter-Mediated Efflux at the Rat Blood–Brain Barrier and Its Implementation into Compound Optimization in Drug Discovery Supplementary Materials

Yunhai Cui, Ralf Lotz, Hermann Rapp, Klaus Klinder, Anneke Himstedt and Achim Sauer

Table S1. Raw data Figure 1 "Correlation of $f_{u,brain}$ and $f_{u,muscle}$." $f_{u,brain}$ = unbound brain concentrations, $f_{u,muscle}$ = unbound muscle concentrations, MW = molecular weight, clogP = calculated logP, a TPSA = total polar surface area, H-AC = number of H acceptors, FSP3 = aliphatic indicator, pKa values are calculated with the software MoKa (Molecular Discovery, Hertfordshire, UK) .

Cpd	Project	$f_{u,brain}$	$f_{u,muscle}$	MW	TPSA	clogP	H-AC	FSP3	cpK_a acidic	cpK_a basic
163	19	0.0084	0.0130	402	69	2.5	4	0.25	12.5	2.2
166	19	0.0062	0.0095	469	75	4.6	4	0.28	11.8	6.0
221	23	0.1000	0.1060	367	93	2.2	5	0.40	10.9	5.0
225	4	0.0099	0.0124	377	68	2.5	2	0.57		6.8
228	4	0.0010	0.0010	413	68	2.8	2	0.57		6.8
229	4	0.4140	0.1860	338	92	0.7	3	0.53		6.5
231	4	0.2352	0.1781	387	86	1.1	4	0.62		7.2
232	4	0.0040	0.0070	420	77	3.4	3	0.44		7.0
238	4	0.0655	0.0662	374	92	1.0	3	0.53		6.5
344	1	0.0208	0.0193	541	119	3.8	8	0.31	9.1	2.2
346	23	0.0273	0.0974	563	101	4.6	6	0.56		5.0
369	23	0.0430	0.0920	507	101	4.3	6	0.54		5.1
386	23	0.0233	0.1227	551	101	4.5	6	0.54		5.0
560	26	0.1592	0.2111	353	93	2.2	6	0.33	7.8	2.9
570	14	0.0954	0.1643	357	95	1.1	6	0.19	10.2	2.9
575	26	0.1526	0.1545	356	80	2.7	5	0.28	9.9	4.1
601	9	0.0282	0.0442	324	46	2.8	4	0.45	11.7	8.2
607	30	0.0009	0.0010	480	71	4.8	5	0.28		6.0
616	6	0.0130	0.0430	379	80	4.2	2	0.24	10.4	8.1
618	27	0.0470	0.0607	449	72	3.3	4	0.25		
619	23	0.0159	0.0220	443	89	3.6	5	0.42		4.2
633	23	0.1160	0.1190	339	91	2.6	4	0.43		4.9
634	27	0.0522	0.0829	444	91	4.4	6	0.29		
635	27	0.0434	0.0470	519	69	5.2	6	0.39	12.8	5.6
642	23	0.0422	0.0830	305	78	1.9	4	0.24	12.0	5.1

Table S2. Raw data Figure 2 "Correlation of $K_{p(br/mu)}$ with (**a**) $K_{p,uu,br(hom)}$ and (**b**) $K_{p,uu,CSF}$.".

Cpd	Project	$K_{p,br/mu}$	$K_{p,uu,br,hom}$	$K_{p,uu,br,CSF}$	MW	TPSA	clogP	H-AC	FSP3
130	27	1.585	1.013	0.860	420	86	3.7	6	0.32
131	27	0.802	1.105	1.948	420	86	3.7	6	0.32
132	27	1.601	1.370	1.261	455	73	4.3	5	0.30
133	27	0.911	0.598	0.479	455	73	4.1	5	0.30
161	19	1.842	0.584	0.998	403	80	2.9	4	0.21
163	19	0.857	0.359	0.424	402	69	2.5	4	0.25
164	19	0.073	0.058	0.222	499	84	2.8	5	0.31
166	19	0.206	0.226	0.603	469	75	4.6	4	0.28
168	19	0.962	0.303	1.210	440	67	4.0	4	0.26
169	19	0.157	0.065	0.234	454	78	4.1	4	0.29
183	28	0.293	0.145	0.351	464	89	2.7	5	0.41
188	19	0.633	0.328	0.427	417	72	2.3	4	0.25
198	28	0.377	0.224	0.336	461	102	1.2	6	0.45
201	28	0.307	0.204	0.431	475	102	1.7	6	0.48
204	19	0.431	0.256	0.282	454	67	3.9	4	0.29
206	27	0.523	0.108	0.223	459	106	3.1	6	0.30
212	19	0.095	0.071	1.663	485	84	4.0	5	0.28
225	4	0.047	0.025	0.078	377	68	2.5	2	0.57
231	4	0.046	0.026	0.090	387	86	1.1	4	0.62
238	4	0.043	0.046	0.239	374	92	1.0	3	0.53
245	4	0.531	0.152	0.294	392	72	3.1	2	0.14
275	23	0.264	0.059	0.126	589	95	3.6	6	0.59
276	23	3.819	0.734	0.384	433	75	3.5	5	0.52
284	23	2.184	0.593	2.186	420	72	4.2	4	0.44
346	23	0.495	0.302	0.408	563	101	4.6	6	0.56
368	23	0.280	0.117	0.385	484	101	3.2	6	0.46
369	23	0.700	0.181	0.381	507	101	4.3	6	0.54
386	23	3.077	0.996	0.728	551	101	4.5	6	0.54
461	38	2.026	1.136	1.025	307	40	4.6	3	0.37
481	38	2.225	1.051	1.000	335	49	4.6	4	0.40
487	38	0.369	0.157	0.355	349	49	4.2	3	0.35
489	38	1.565	0.723	0.708	308	61	2.9	3	0.47
499	27	1.275	0.185	0.471	438	86	3.5	6	0.32
500	27	0.654	0.169	0.255	434	81	3.0	5	0.33
501	27	0.731	0.903	1.575	452	81	3.2	5	0.33
502	27	0.925	0.648	1.067	406	106	3.2	7	0.29
503	27	1.250	1.319	1.194	407	79	4.0	5	0.27
505	27	0.767	1.238	1.208	404	97	4.1	6	0.32
506	27	0.545	1.523	1.550	429	97	4.3	5	0.25
508	38	0.733	0.226	0.475	336	62	3.9	5	0.42
525	26	1.835	0.394	0.526	297	90	1.4	5	0.47
540	26	0.917	0.478	0.673	288	50	1.6	3	0.33
555	13	0.458	0.272	2.448	427	65	4.0	3	0.22
556	19	1.998	0.958	1.716	445	65	3.9	3	0.22
557	19	0.686	0.152	0.306	402	72	3.2	4	0.21
558	19	0.145	0.076	0.188	390	80	1.8	4	0.21
559	19	1.665	1.247	0.603	380	52	5.0	3	0.22
560	26	0.467	0.294	0.538	353	93	2.2	6	0.33
570	14	0.661	0.201	0.226	357	95	1.1	6	0.19
575	26	0.226	0.113	0.203	356	80	2.7	5	0.28
601	9	0.056	0.028	0.174	324	46	2.8	4	0.45
618	27	0.328	0.336	0.488	449	72	3.3	4	0.25
619	23	1.496	0.297	0.603	443	89	3.6	5	0.42
623	39	1.504	0.671	0.627	333	49	3.2	2	0.43
628	39	1.154	1.409	9.018	345	25	4.9	1	0.45
634	27	0.758	1.192	0.928	444	91	4.4	6	0.29
635	27	0.286	0.804	2.511	519	69	5.2	6	0.39
642	23	1.440	0.958	2.035	305	78	1.9	4	0.24
655	13	5.994	5.515	3.225	194	48	1.5	1	0.30
658	38	2.347	0.990	0.670	329	79	3.2	4	0.44
659	38	1.378	2.146	1.071	348	73	3.9	5	0.35
672	26	0.317	0.851	0.573	327	64	1.8	4	0.40
677	9	0.180	0.062	1.035	314	122	1.6	5	0.07

Table S2. continued

Cpd	Project	$K_{p,br/mu}$	$K_{p,uu,br,hom}$	$K_{p,uu,br,CSF}$	MW	TPSA	clogP	H-AC	FSP3
682	9	0.032	0.018	0.053	456	73	1.0	5	0.36

Table S3. Raw data Figure 3 "Correlation of $K_{p,br/mu}$ values obtained following intravenous and oral dosing for a set of 29 compounds."

Cpd	Project	i.v. $K_{p,br/mu}$	p.o. $K_{p,br/mu}$
5	29	0.065	0.088
15	21	0.199	0.231
17	21	0.015	0.023
18	21	0.505	0.647
20	21	0.122	0.160
21	21	0.136	0.132
22	29	0.188	0.156
23	21	0.185	0.176
24	23	0.265	0.739
32	23	0.127	0.176
34	23	0.442	1.465
36	23	1.022	3.501
43	23	0.185	0.652
44	23	0.439	0.334
45	23	0.159	0.042
48	23	0.245	0.437
108	28	1.652	1.051
222	16	0.115	0.086
247	34	0.032	0.039
248	42	0.314	0.281
300	28	0.816	0.631
305	28	1.352	1.498
335	34	0.037	0.042
371	28	0.668	1.477
449	27	0.728	0.637
466	28	0.717	1.603
606	13	0.302	0.351
607	20	0.326	0.801
610	39	0.031	0.094

Table S4. Raw data Figure 4 "In vitro – in vivo correlation of MDR1 P-gp efflux and $K_{p,br/mu}$." Subgroup 1 depicted in red, Subgroup 2 in blue

Cpd	Project	Route	$MDCK_{Pgp\_10\_Efflux}$	$K_{p,br/mu}$
1	23	p.o.	0.568	1.045
2	34	p.o.	1.441	0.107
3	34	i.v.	0.565	0.949
4	29	p.o.	2.219	0.290
5	29	i.v.	18.810	0.065
5	29	p.o.	18.810	0.088
6	21	i.v.	23.110	0.086
7	21	p.o.	19.430	0.084
8	21	p.o.	31.920	0.061
9	21	p.o.	7.193	0.680
10	21	p.o.	4.064	0.474
11	21	p.o.	10.820	0.265
12	21	p.o.	4.089	1.051
13	21	p.o.	15.120	0.170
14	21	p.o.	13.250	0.137
15	21	i.v.	4.610	0.199
15	21	p.o.	4.610	0.231
16	21	i.v.	14.040	0.029
17	21	i.v.	32.790	0.015
17	21	p.o.	32.790	0.023
18	21	i.v.	1.214	0.505
18	21	p.o.	1.214	0.647
19	21	i.v.	31.620	0.035
20	21	i.v.	12.530	0.122
20	21	p.o.	12.530	0.160
21	21	i.v.	4.345	0.136
21	21	p.o.	4.345	0.132
22	29	i.v.	3.512	0.188
22	29	p.o.	3.512	0.156
23	21	i.v.	6.528	0.185
23	21	p.o.	6.528	0.176
24	23	i.v.	0.904	0.265
24	23	p.o.	0.904	0.739
25	23	i.v.	2.251	0.577
26	23	i.v.	0.528	1.870
27	21	p.o.	13.410	0.255
28	21	p.o.	3.349	0.537
29	21	p.o.	13.460	0.222
30	21	p.o.	37.310	0.192
31	23	i.v.	0.527	1.370
32	23	i.v.	4.556	0.127
32	23	p.o.	4.556	0.176
33	34	i.v.	0.614	1.840
34	23	i.v.	0.492	0.442
34	23	p.o.	0.492	1.465
35	23	i.v.	11.210	0.114
36	23	i.v.	0.548	1.022
36	23	p.o.	0.548	3.501
37	23	i.v.	9.386	0.364
38	23	i.v.	9.471	0.076
39	23	i.v.	0.656	1.142
40	23	i.v.	0.547	0.787
41	23	i.v.	0.764	0.607
42	23	i.v.	1.736	0.497
43	23	i.v.	0.697	0.185
43	23	p.o.	0.697	0.652
44	23	i.v.	0.826	0.439
44	23	p.o.	0.826	0.334
45	23	i.v.	14.090	0.159
45	23	p.o.	14.090	0.042
46	23	i.v.	0.423	0.354
47	23	i.v.	4.907	0.055
48	23	i.v.	0.881	0.245
48	23	p.o.	0.881	0.437
49	21	p.o.	26.530	0.184
50	33	p.o.	27.950	0.055
51	21	p.o.	23.320	0.114
52	21	p.o.	1.505	0.160
53	21	p.o.	6.463	0.378
54	28	p.o.	1.315	0.467
55	21	p.o.	10.500	0.315
56	33	i.v.	46.510	0.064
57	33	i.v.	26.860	0.078
58	33	p.o.	25.110	0.085
59	21	p.o.	6.386	0.463
60	33	p.o.	36.000	0.048
61	21	p.o.	5.266	0.254
62	21	p.o.	3.242	0.417
63	21	p.o.	25.440	0.062
64	21	p.o.	4.113	0.228
65	21	p.o.	15.630	0.075
66	21	p.o.	6.857	0.056
67	28	p.o.	0.600	1.633
68	28	p.o.	0.586	2.356
69	28	p.o.	0.718	2.996
70	28	p.o.	2.161	0.658
71	28	p.o.	1.841	0.308
72	28	i.v.	1.039	0.589
73	28	p.o.	2.548	0.430
74	28	p.o.	1.248	0.591
75	28	p.o.	1.638	0.999
76	28	p.o.	0.682	2.718
77	28	p.o.	1.922	0.664
78	33	p.o.	38.455	0.050
79	21	p.o.	2.666	1.152
80	21	p.o.	11.450	0.281
81	21	p.o.	3.647	0.234
82	21	p.o.	21.900	0.062
83	28	p.o.	0.523	1.970
84	28	p.o.	0.576	2.702
85	28	p.o.	1.514	0.950
86	28	p.o.	1.660	0.592
87	28	p.o.	0.689	1.616
88	28	p.o.	1.400	0.446
89	28	p.o.	0.834	1.967
90	33	p.o.	4.679	0.027
91	28	p.o.	0.617	0.120
92	28	p.o.	1.852	0.496
93	28	i.v.	1.269	0.254
94	28	p.o.	0.863	0.271
95	28	p.o.	0.582	1.964
96	28	p.o.	1.518	0.430
97	28	p.o.	0.681	0.978
98	28	p.o.	0.988	0.488
99	28	p.o.	0.511	0.956
100	28	p.o.	1.178	0.298
101	28	p.o.	1.168	0.388
102	28	p.o.	0.519	2.305
103	28	p.o.	0.475	2.537
104	28	i.v.	0.745	0.187
105	28	p.o.	0.664	0.862
106	28	p.o.	0.541	1.194

Table S4. continued

Cpd	Project	Route	$MDCK_{Pgp\_10\_Efflux}$	$K_{p,br/mu}$	Cpd	Project	Route	$MDCK_{Pgp\_10\_Efflux}$	$K_{p,br/mu}$
107	28	p.o.	0.587	1.741	166	19	p.o.	3.930	0.206
108	28	i.v.	0.819	1.652	167	19	p.o.	1.036	0.476
108	28	p.o.	0.819	1.051	168	19	p.o.	0.345	0.962
109	28	p.o.	0.692	1.909	169	19	p.o.	0.960	0.157
110	28	i.v.	4.410	0.151	170	19	p.o.	0.470	1.185
111	28	i.v.	3.669	0.196	171	19	p.o.	2.190	0.062
112	28	p.o.	8.267	0.315	172	27	p.o.	1.009	0.686
113	28	p.o.	3.614	0.223	173	27	p.o.	1.334	0.476
114	28	p.o.	4.884	0.257	174	27	p.o.	2.542	0.384
115	28	i.v.	1.504	0.412	175	27	p.o.	1.275	1.371
116	28	i.v.	2.981	0.302	176	27	p.o.	1.178	0.648
117	28	p.o.	3.658	0.257	177	27	p.o.	1.024	1.161
118	28	i.v.	15.020	0.182	178	27	p.o.	0.619	1.247
119	28	i.v.	8.356	0.086	179	27	p.o.	3.824	0.294
119	28	i.v.	8.356	0.120	180	27	p.o.	0.943	1.124
120	28	i.v.	2.076	0.593	181	27	p.o.	1.515	0.594
121	21	p.o.	2.179	0.345	182	27	p.o.	2.611	0.844
122	21	p.o.	3.774	0.181	183	28	p.o.	0.949	0.293
123	28	p.o.	8.361	0.183	184	28	p.o.	1.427	0.397
124	28	i.v.	6.865	0.089	185	28	p.o.	2.996	0.207
125	28	p.o.	7.544	0.152	186	28	p.o.	2.132	0.739
126	28	p.o.	6.337	0.142	187	27	p.o.	0.707	0.826
127	28	p.o.	0.664	0.100	189	19	p.o.	0.990	0.632
128	28	i.v.	2.032	0.446	190	19	p.o.	1.640	0.291
129	33	p.o.	1.026	2.268	191	28	p.o.	1.074	0.722
130	27	p.o.	0.769	1.585	192	28	p.o.	1.976	0.616
131	27	i.v.	0.843	0.802	193	19	p.o.	1.297	0.294
132	27	p.o.	0.631	1.601	194	19	p.o.	0.847	0.679
133	27	p.o.	0.641	0.911	195	19	p.o.	1.910	0.154
134	27	p.o.	1.511	0.495	196	19	p.o.	0.494	1.094
135	27	p.o.	0.808	1.401	197	19	p.o.	3.308	0.225
136	27	p.o.	0.667	0.919	198	28	p.o.	2.895	0.377
137	28	i.v.	0.702	1.252	199	28	p.o.	1.359	0.297
138	28	p.o.	0.639	1.226	200	28	p.o.	1.609	0.344
139	28	p.o.	0.650	1.325	201	28	p.o.	2.552	0.307
140	28	p.o.	0.637	1.449	202	19	p.o.	2.965	0.241
141	28	p.o.	0.605	0.381	203	28	p.o.	3.491	0.147
142	28	p.o.	0.947	0.633	204	19	p.o.	0.830	0.431
143	28	i.v.	0.543	1.260	205	27	p.o.	2.995	0.367
144	28	p.o.	0.613	1.341	206	27	i.v.	1.128	0.523
145	28	p.o.	0.640	1.183	207	27	i.v.	0.772	0.650
146	28	p.o.	0.799	0.600	208	27	p.o.	3.555	0.290
147	28	p.o.	0.628	0.964	209	27	p.o.	1.276	0.599
148	33	p.o.	4.785	0.121	210	19	p.o.	1.317	0.123
149	33	p.o.	1.228	1.328	211	19	p.o.	1.570	0.314
150	33	p.o.	4.159	0.118	212	19	p.o.	1.270	0.095
151	33	p.o.	0.964	1.544	213	19	p.o.	0.990	0.746
152	28	p.o.	8.150	0.322	214	10	p.o.	3.010	0.562
153	27	p.o.	1.529	0.640	215	10	p.o.	8.026	0.172
154	27	i.v.	5.138	0.225	216	10	p.o.	17.340	0.148
155	27	i.v.	1.978	0.458	217	10	p.o.	16.400	0.162
156	27	i.v.	0.831	0.422	218	37	p.o.	3.929	0.046
157	27	p.o.	1.399	0.194	219	28	p.o.	17.370	0.044
158	27	p.o.	2.858	0.370	220	23	p.o.	0.667	0.373
159	28	p.o.	1.297	0.605	221	23	p.o.	1.061	0.719
160	19	p.o.	0.810	0.874	222	16	i.v.	26.350	0.115
161	19	p.o.	0.530	1.842	222	16	p.o.	26.350	0.086
162	19	p.o.	1.033	0.217	223	23	p.o.	0.532	1.061
163	19	p.o.	0.700	0.857	224	20	p.o.	2.266	0.129
164	19	p.o.	5.050	0.073	225	4	p.o.	61.980	0.047
165	19	p.o.	1.120	0.339	226	25	p.o.	28.460	0.048

Table S4. continued

Cpd	Project	Route	$MDCK_{Pgp\_10\_Efflux}$	$K_{p,br/mu}$
227	25	p.o.	10.560	0.056
228	4	p.o.	34.340	0.074
229	4	p.o.	50.530	0.049
230	20	i.v.	0.661	0.464
231	4	p.o.	35.150	0.046
232	4	p.o.	64.350	0.044
233	26	p.o.	1.470	0.902
234	4	p.o.	19.820	0.107
235	20	i.v.	8.140	0.186
236	33	i.p.l	0.408	2.742
237	18	p.o.	0.613	1.750
238	4	p.o.	59.790	0.043
239	41	p.o.	3.718	0.175
240	41	p.o.	0.513	2.648
241	12	p.o.	1.396	0.805
242	20	p.o.	0.601	0.384
243	20	p.o.	0.512	1.273
244	20	p.o.	9.442	0.063
245	4	p.o.	3.675	0.531
246	4	p.o.	4.263	0.575
247	34	i.v.	26.770	0.032
247	34	p.o.	26.770	0.039
248	42	i.v.	3.144	0.314
248	42	p.o.	3.144	0.281
249	4	p.o.	20.190	0.059
250	4	p.o.	23.930	0.038
251	42	p.o.	2.109	0.331
252	42	p.o.	1.645	0.247
253	20	p.o.	0.490	1.128
254	33	p.o.	26.430	0.027
255	11	i.v.	5.942	0.391
256	42	p.o.	0.648	2.294
257	20	p.o.	13.680	0.197
258	20	p.o.	1.209	0.258
259	20	p.o.	0.720	0.527
260	20	p.o.	1.019	0.519
261	33	p.o.	14.310	0.145
262	33	p.o.	1.084	0.165
263	20	p.o.	4.039	0.088
264	20	p.o.	0.530	0.339
265	20	p.o.	0.473	0.100
266	20	p.o.	1.190	0.179
267	20	p.o.	1.170	0.038
268	20	p.o.	1.010	0.256
269	20	p.o.	0.720	0.256
270	20	p.o.	1.000	0.239
271	20	p.o.	0.720	0.210
272	20	p.o.	2.370	0.059
273	4	p.o.	74.030	0.069
274	34	p.o.	9.265	0.053
275	23	p.o.	8.193	0.264
276	23	p.o.	0.483	3.819
277	23	p.o.	3.056	0.320
278	10	p.o.	2.525	0.378
279	28	i.v.	0.575	0.269
280	20	p.o.	0.822	0.362
281	20	p.o.	1.222	0.760
282	20	p.o.	7.940	0.080
283	23	p.o.	0.862	0.753
284	23	p.o.	0.638	2.184
285	20	p.o.	1.064	0.379
286	20	p.o.	1.166	0.322
287	20	p.o.	1.145	0.418
288	20	p.o.	2.050	0.227
289	20	p.o.	0.583	0.387
290	20	p.o.	0.650	0.273
291	20	p.o.	0.620	0.989
292	20	p.o.	0.940	0.125
293	20	p.o.	1.370	0.095
294	20	p.o.	2.036	0.298
295	15	p.o.	0.882	0.691
296	23	p.o.	0.927	1.128
297	28	i.v.	24.130	0.053
298	28	p.o.	0.506	2.150
299	28	p.o.	1.586	0.963
300	28	i.v.	1.594	0.816
300	28	p.o.	1.594	0.631
301	28	p.o.	1.783	0.952
302	28	p.o.	2.149	0.543
303	28	p.o.	0.562	0.673
304	28	i.v.	0.361	3.204
305	28	i.v.	0.671	1.352
305	28	p.o.	0.671	1.498
306	28	p.o.	0.742	0.488
307	20	p.o.	0.510	0.198
308	20	p.o.	0.640	0.206
309	20	p.o.	0.436	0.247
310	20	p.o.	0.474	0.089
311	15	p.o.	1.389	0.379
312	4	p.o.	2.303	1.158
313	4	p.o.	13.690	0.090
314	20	p.o.	2.247	0.324
315	20	p.o.	3.421	0.161
316	20	p.o.	0.678	0.268
317	23	p.o.	3.239	0.639
318	15	p.o.	1.023	0.859
319	15	p.o.	3.504	0.134
320	15	p.o.	3.750	0.098
321	15	p.o.	7.618	0.108
322	15	p.o.	11.670	0.073
323	15	p.o.	2.619	0.408
324	15	p.o.	1.473	0.722
325	15	p.o.	5.445	0.164
326	15	p.o.	18.260	0.072
327	15	p.o.	0.792	1.423
328	15	p.o.	0.588	1.573
329	15	p.o.	1.138	0.566
330	20	p.o.	1.122	0.154
331	20	p.o.	0.570	0.151
332	23	p.o.	1.787	0.251
333	4	p.o.	7.683	0.115
334	23	p.o.	0.604	2.917
335	34	i.v.	45.520	0.037
335	34	p.o.	45.520	0.042
336	15	p.o.	0.671	0.232
337	15	p.o.	19.610	0.019
338	15	p.o.	0.990	0.359
339	15	p.o.	8.713	0.100
340	15	p.o.	0.665	1.311
341	15	p.o.	6.387	0.075
342	15	p.o.	6.973	0.073
343	23	p.o.	0.577	1.977

Table S4. continued

Cpd	Project	Route	MDCK$_{Pgp_10_Efflux}$	$K_{p,br/mu}$
344	1	p.o.	68.380	0.096
345	25	p.o.	0.594	0.431
346	23	p.o.	1.166	0.495
347	23	p.o.	3.462	0.997
348	27	i.v.	0.664	0.802
349	23	p.o.	0.608	0.497
350	23	p.o.	3.453	0.393
351	25	p.o.	3.312	0.150
352	23	p.o.	0.594	2.362
353	15	p.o.	2.925	0.181
354	15	p.o.	37.860	0.182
355	15	p.o.	3.277	0.354
356	23	p.o.	0.545	2.646
357	38	p.o.	1.699	0.297
358	38	p.o.	1.227	0.283
359	38	p.o.	1.936	0.263
360	38	p.o.	5.504	0.255
361	23	p.o.	1.353	1.240
362	23	p.o.	1.140	1.702
363	23	p.o.	0.740	1.345
364	15	p.o.	7.552	0.112
365	15	p.o.	0.929	0.368
366	15	p.o.	4.776	0.083
367	15	p.o.	5.642	0.046
368	23	p.o.	0.696	0.280
369	23	p.o.	2.994	0.700
370	23	p.o.	2.682	1.160
371	28	i.v.	0.581	0.668
371	28	p.o.	0.581	1.477
372	15	p.o.	10.480	0.077
373	15	p.o.	0.750	0.991
374	15	p.o.	1.047	0.473
375	15	p.o.	0.848	0.499
376	23	p.o.	1.119	2.088
377	15	p.o.	1.397	0.611
378	15	p.o.	3.268	0.172
379	38	p.o.	0.630	1.212
380	23	p.o.	3.940	1.567
381	23	p.o.	3.396	0.477
382	38	p.o.	4.090	0.798
383	34	i.v.	26.880	0.054
384	28	i.v.	0.733	0.437
385	39	s.c.	1.265	0.681
386	23	p.o.	1.566	3.077
387	23	p.o.	1.002	0.957
388	23	p.o.	0.759	0.809
389	18	p.o.	8.697	0.201
390	23	p.o.	2.232	1.205
391	38	p.o.	1.610	0.333
392	34	i.v.	29.710	0.039
393	23	p.o.	0.865	0.750
394	15	p.o.	2.660	0.193
395	15	p.o.	0.928	0.283
396	15	p.o.	0.982	0.236
397	15	p.o.	0.626	0.954
398	15	p.o.	1.489	0.622
399	27	i.v.	6.760	0.233
400	38	p.o.	1.373	0.443
401	38	p.o.	1.926	0.118
402	27	i.v.	1.162	0.234
403	27	i.v.	1.388	0.368
404	27	i.v.	0.849	0.643
405	4	p.o.	47.660	0.062
406	15	p.o.	1.301	0.140
407	15	p.o.	0.687	0.560
408	15	p.o.	3.251	0.090
409	15	p.o.	2.280	0.285
410	15	p.o.	8.531	0.054
411	15	p.o.	1.075	0.192
412	15	p.o.	1.892	0.186
413	15	p.o.	28.720	0.101
414	27	i.v.	2.393	0.472
415	27	p.o.	3.017	0.452
416	27	p.o.	1.075	0.649
417	27	i.v.	0.569	1.050
418	27	intraperitoneal	0.700	1.331
419	27	i.v.	1.426	0.723
420	27	i.v.	2.310	0.391
421	15	p.o.	12.080	0.065
422	15	p.o.	4.351	0.170
423	15	p.o.	0.639	1.674
424	15	p.o.	1.067	0.202
425	15	p.o.	0.782	1.234
426	15	p.o.	1.080	0.680
427	15	p.o.	1.303	0.507
428	15	p.o.	6.807	0.045
429	15	p.o.	0.895	0.365
430	15	p.o.	0.955	0.735
431	15	p.o.	0.888	0.377
432	15	p.o.	0.920	0.616
433	15	p.o.	12.250	0.099
434	28	i.v.	0.548	0.187
435	23	p.o.	1.198	1.516
436	27	i.v.	7.635	0.123
437	26	p.o.	4.626	0.120
438	26	p.o.	0.620	1.205
439	29	p.o.	4.154	0.173
440	26	p.o.	1.268	0.212
441	26	p.o.	1.100	0.086
442	26	p.o.	1.180	0.192
443	26	p.o.	3.730	0.207
444	39	p.o.	4.610	0.437
445	38	p.o.	7.319	0.182
446	38	p.o.	14.950	0.062
447	29	p.o.	0.613	1.526
448	26	p.o.	9.320	0.114
449	27	i.v.	0.827	0.728
449	27	p.o.	0.827	0.637
450	27	p.o.	0.634	0.710
451	27	p.o.	2.353	0.573
452	27	i.v.	8.017	0.049
453	36	p.o.	0.649	0.364
454	15	p.o.	9.761	0.107
455	15	p.o.	6.089	0.019
456	15	p.o.	1.911	0.300
457	15	p.o.	1.218	0.429
458	27	i.v.	4.835	0.156
459	36	p.o.	1.396	0.488
460	39	p.o.	0.750	1.328
461	38	p.o.	0.549	2.026
462	38	p.o.	0.612	2.063
463	39	p.o.	0.654	1.113

Table S4. continued

Cpd	Project	Route	$MDCK_{Pgp\_10\_Efflux}$	$K_{p,br/mu}$
464	38	p.o.	0.667	1.035
465	38	p.o.	0.538	2.119
466	28	i.v.	0.709	0.718
466	28	p.o.	0.709	1.603
467	38	p.o.	0.625	1.486
468	15	p.o.	21.870	0.095
469	15	p.o.	0.558	1.123
470	15	p.o.	0.666	1.186
471	15	p.o.	0.737	1.176
472	15	p.o.	1.344	0.455
473	28	p.o.	0.616	2.158
474	38	p.o.	0.739	1.254
475	26	p.o.	4.200	0.108
476	26	p.o.	4.110	0.087
477	26	p.o.	1.496	0.405
478	26	p.o.	1.400	0.401
479	38	p.o.	0.602	2.959
480	38	p.o.	0.624	1.517
481	38	p.o.	0.590	2.225
482	38	p.o.	0.890	1.220
483	38	p.o.	3.084	0.473
484	39	p.o.	0.613	1.629
485	15	p.o.	11.480	0.050
486	38	p.o.	1.847	0.504
487	38	p.o.	3.156	0.369
488	38	p.o.	0.741	0.330
489	38	p.o.	1.401	1.565
490	38	p.o.	4.000	0.168
491	38	p.o.	3.407	0.376
492	38	p.o.	0.706	0.602
493	38	p.o.	0.731	1.571
494	38	p.o.	0.630	1.455
495	15	p.o.	2.862	0.181
496	38	p.o.	0.578	1.434
497	27	i.v.	0.717	0.294
498	27	p.o.	0.983	0.797
499	27	p.o.	0.735	1.275
500	27	p.o.	1.186	0.654
501	27	i.v.	0.746	0.731
502	27	p.o.	0.969	0.925
503	27	p.o.	0.718	1.250
504	27	p.o.	0.831	0.632
505	27	p.o.	0.915	0.767
506	27	p.o.	1.266	0.545
507	38	p.o.	0.596	1.444
508	38	p.o.	1.217	0.733
509	38	p.o.	0.841	0.930
510	15	p.o.	1.830	0.331
511	15	p.o.	0.881	0.936
512	15	p.o.	0.551	0.327
513	15	p.o.	2.513	0.159
514	15	p.o.	1.383	0.338
515	15	p.o.	1.159	0.328
516	27	p.o.	4.400	0.133
517	15	p.o.	0.710	0.747
518	15	p.o.	4.579	0.125
519	15	p.o.	1.432	0.384
520	15	p.o.	2.347	0.463
521	15	p.o.	1.599	0.319
522	26	p.o.	1.480	0.134
523	26	p.o.	1.360	0.147
524	26	p.o.	0.648	1.505
525	26	p.o.	0.310	1.835
526	38	p.o.	0.513	0.985
527	38	p.o.	1.739	0.291
528	38	p.o.	2.975	0.304
529	38	p.o.	3.077	0.522
530	27	p.o.	0.793	0.915
531	27	p.o.	0.706	1.104
532	27	p.o.	0.650	1.512
533	27	p.o.	0.679	1.985
534	27	i.v.	0.589	1.121
535	27	i.v.	0.596	1.369
536	38	p.o.	2.177	0.244
537	38	p.o.	2.870	0.192
538	38	p.o.	0.752	1.071
539	26	p.o.	0.570	2.574
540	26	p.o.	1.150	0.917
541	27	p.o.	3.940	0.140
542	27	p.o.	1.703	1.172
543	27	i.v.	1.289	1.185
544	27	p.o.	2.661	1.504
545	38	p.o.	1.032	1.059
546	38	p.o.	1.042	0.777
547	38	p.o.	0.730	1.243
548	38	p.o.	0.543	1.992
549	26	p.o.	1.980	0.171
550	38	p.o.	1.113	1.777
551	27	i.v.	1.899	0.505
552	27	p.o.	1.583	0.287
553	2	i.v.	5.959	0.135
554	22	p.o.	0.560	0.720
555	13	p.o.	0.537	0.458
556	19	p.o.	0.450	1.998
557	19	p.o.	0.880	0.686
558	19	p.o.	2.020	0.145
559	19	p.o.	0.410	1.665
560	26	p.o.	1.550	0.467
561	26	p.o.	5.800	0.163
562	26	p.o.	6.020	0.144
563	26	p.o.	0.930	0.540
564	26	p.o.	3.096	0.173
565	22	p.o.	4.405	0.279
566	22	p.o.	3.238	0.163
567	14	p.o.	2.597	0.166
568	14	p.o.	0.609	1.639
569	14	p.o.	1.924	0.230
570	14	p.o.	2.388	0.661
571	14	p.o.	0.975	1.285
572	14	p.o.	3.029	0.143
573	40	p.o.	0.586	0.732
574	22	p.o.	0.670	0.358
575	26	p.o.	1.690	0.226
576	26	p.o.	2.640	0.266
577	26	p.o.	1.430	0.251
578	22	p.o.	2.110	0.152
579	14	p.o.	7.796	0.150
580	14	p.o.	4.171	0.094
581	14	p.o.	2.434	0.192
582	26	p.o.	0.430	1.222
583	26	p.o.	0.730	1.384
584	14	p.o.	2.751	0.589

Table S4. continued

Cpd	Project	Route	$MDCK_{Pgp\_10\_Efflux}$	$K_{p,br/mu}$
585	22	p.o.	0.840	0.441
586	40	p.o.	0.860	1.279
587	24	p.o.	3.000	0.347
588	24	p.o.	0.660	1.281
589	24	p.o.	0.620	0.705
590	34	p.o.	2.851	0.090
591	38	s.c.	1.455	0.378
592	22	p.o.	0.990	0.331
593	24	p.o.	1.000	0.487
594	24	p.o.	1.420	0.282
595	24	p.o.	0.800	0.099
596	24	p.o.	1.120	0.444
597	24	p.o.	0.970	0.422
598	24	p.o.	1.510	0.350
599	40	p.o.	0.977	1.875
600	24	p.o.	1.417	0.341
601	9	i.v.	26.081	0.056
602	9	p.o.	0.597	1.437
603	41	p.o.	0.523	0.780
604	42	i.p.l	5.453	0.279
605	20	p.o.	0.370	1.288
606	13	p.o.	2.320	0.351
606	13	i.v.	2.320	0.302
607	20	i.v.	0.343	0.328
607	20	p.o.	0.340	0.801
608	41	p.o.	0.632	0.588
609	20	i.v.	0.551	0.285
610	39	p.o.	15.710	0.094
610	39	i.v.	15.710	0.031
611	41	p.o.	0.584	1.262
612	29	p.o.	0.466	2.107
613	20	i.v.	1.334	1.261
614	33	i.p.	23.900	0.085
615	41	p.o.	0.825	0.685
616	6	p.o.	1.662	0.294
617	27	i.v.	0.625	0.398
618	27	p.o.	0.685	0.328
619	23	p.o.	0.641	1.496
620	4	p.o.	4.357	0.239
621	13	i.p.	7.213	0.101
622	20	p.o.	0.370	1.301
623	39	p.o.	0.621	1.504
624	13	p.o.	0.443	2.071
625	23	i.v.	0.706	0.433
626	17	p.o.	0.526	1.508
627	5	p.o.	0.583	1.572
628	39	s.c.	0.555	1.154
629	27	p.o.	0.853	0.538
630	7	i.p.	0.620	0.583
631	27	i.v.	1.095	0.399
632	27	i.p.	0.636	2.308
633	23	p.o.	0.639	1.528
634	27	p.o.	0.752	0.758
635	27	p.o.	0.945	0.286
636	39	p.o.	3.409	0.430
637	39	p.o.	0.602	1.681
638	4	p.o.	24.180	0.056
639	4	p.o.	17.410	0.031
640	17	p.o.	5.688	0.166
641	17	p.o.	0.534	1.687
642	23	p.o.	1.000	1.440
643	26	p.o.	2.490	0.215
644	8	p.o.	0.547	1.578
645	17	p.o.	0.722	1.025
646	13	p.o.	1.341	0.402
647	13	p.o.	6.103	0.201
648	13	p.o.	11.380	0.046
649	35	p.o.	1.010	0.627
650	24	p.o.	3.530	0.120
651	36	p.o.	2.957	0.123
652	38	p.o.	0.534	3.045
652	38	s.c.	0.534	3.622
653	15	p.o.	21.270	0.041
654	5	p.o.	0.608	0.559
655	13	p.o.	0.562	5.994
656	38	p.o.	0.572	1.567
657	13	i.p.	0.727	2.070
658	38	p.o.	0.818	2.347
659	38	p.o.	0.486	1.378
660	26	i.p.	0.860	1.928
661	13	p.o.	0.456	1.151
662	13	p.o.	1.055	0.519
663	13	p.o.	0.427	0.581
664	15	p.o.	13.560	0.128
665	24	p.o.	1.174	1.229
666	9	p.o.	2.808	0.110
667	28	p.o.	0.551	2.435
668	28	p.o.	1.231	1.280
669	26	i.p.	0.682	1.409
670	28	i.v.	1.529	0.581
671	28	p.o.	0.545	1.683
672	26	i.p.	2.239	0.317
673	28	i.v.	1.435	0.326
674	28	p.o.	3.295	0.260
675	28	i.v.	2.986	0.600
676	31	p.o.	1.241	0.095
677	9	i.v.	0.685	0.180
678	9	p.o.	1.623	0.508
679	9	i.v.	0.838	0.603
680	24	i.p.	0.259	5.141
681	32	p.o.	5.489	0.063
682	9	i.v.	21.945	0.032

Table S5. Raw data Figure 5 "Correlation of $K_{p,br/mu}$ with in vitro efflux measured in MDCK-MDR1 cells for compounds of three research projects (N=48)."

Cpd	Project	$K_{p,br/mu}$	$MDCK_{Pgp\_10\_Efflux}$	$MDCK_{Pgp\_1\_Efflux}$
160	19	0.874	0.810	0.850
161	19	1.842	0.530	0.350
163	19	0.857	0.700	1.120
164	19	0.073	5.050	6.290
165	19	0.339	1.120	1.450
166	19	0.206	3.930	5.330
168	19	0.962	0.345	0.592
169	19	0.157	0.960	2.630
170	19	1.185	0.470	0.480
171	19	0.062	2.190	5.430
189	19	0.632	0.990	1.110
190	19	0.291	1.640	2.680
195	19	0.154	1.910	4.660
204	19	0.431	0.830	1.220
211	19	0.314	1.570	3.800
212	19	0.095	1.270	2.200
213	19	0.746	0.990	1.360
259	20	0.527	0.720	2.500
264	20	0.339	0.530	2.230
266	20	0.179	1.190	3.390
267	20	0.038	1.170	5.380
268	20	0.256	1.010	6.570
269	20	0.256	0.720	5.160
270	20	0.239	1.000	6.760
271	20	0.210	0.720	0.730
272	20	0.059	2.370	4.950
282	20	0.080	7.940	12.080
288	20	0.227	2.050	6.200
290	20	0.273	0.650	3.540
291	20	0.989	0.620	4.280
292	20	0.125	0.940	4.100
293	20	0.095	1.370	5.290
307	20	0.198	0.510	0.580
308	20	0.206	0.640	3.430
330	20	0.154	1.122	8.429
331	20	0.151	0.570	1.260
554	22	0.720	0.560	0.580
556	19	1.998	0.450	0.590
557	19	0.686	0.880	0.880
558	19	0.145	2.020	2.810
559	19	1.665	0.410	0.380
574	22	0.358	0.670	2.280
578	22	0.152	2.110	3.240
585	22	0.441	0.840	1.590
592	22	0.331	0.990	2.170
605	20	1.288	0.370	0.610
607	20	0.801	0.340	0.770
622	20	1.301	0.370	0.550

Table S6. Raw data Figure 6 "Correlation of $K_{p,br/mu}$ with in vitro efflux measured in MDCK-MDR1 and MDCK-BCRP cells for compounds from two research projects (N = 39)."

Cpd	Project	$K_{p,br/mu}$	$MDCK_{Pgp\_10\_Efflux}$	$MDCK_{BCRP\_10\_Efflux}$	Equation based Efflux
233	26	0.902	1.470	9.080	2.817
438	26	1.205	0.620	0.840	0.597
441	26	0.086	1.100	14.800	3.400
442	26	0.192	1.180	19.830	4.319
443	26	0.207	3.730	26.620	8.003
448	26	0.114	9.320	36.730	15.271
475	26	0.108	4.200	47.580	11.964
476	26	0.087	4.110	53.400	12.841
478	26	0.401	1.400	2.000	1.566
522	26	0.134	1.480	13.910	3.636
523	26	0.147	1.360	6.730	2.317
525	26	1.835	0.310	0.810	0.276
539	26	2.574	0.570	0.420	0.474
540	26	0.917	1.150	0.760	1.111
549	26	0.171	1.980	15.550	4.409
560	26	0.467	1.550	12.730	3.505
561	26	0.163	5.800	23.750	9.588
562	26	0.144	6.020	15.210	8.387
563	26	0.540	0.930	7.220	1.966
575	26	0.226	1.690	2.900	2.008
576	26	0.266	2.640	22.320	6.195
577	26	0.251	1.430	17.060	4.105
582	26	1.222	0.430	0.710	0.379
583	26	1.384	0.730	0.570	0.654
587	24	0.347	3.000	4.270	3.545
588	24	1.281	0.660	0.800	0.625
589	24	0.705	0.620	0.990	0.613
593	24	0.487	1.000	2.000	1.170
594	24	0.282	1.420	2.240	1.627
595	24	0.099	0.800	16.690	3.420
596	24	0.444	1.120	3.740	1.578
597	24	0.422	0.970	4.890	1.624
598	24	0.350	1.510	5.940	2.333
643	26	0.215	2.490	5.010	3.161
650	24	0.120	3.530	1.320	3.579
660	26	1.928	0.860	0.608	0.795
669	26	1.409	0.682	0.696	0.631
672	26	0.317	2.239	0.731	2.194
680	24	5.141	0.259	0.740	0.216

Table S7: Raw data Figure S2 "In vitro – in vivo correlation of MDCK P-gp efflux and mouse $K_{p,br/mu}$."

Cpd	Project	Route	$MDCK_{Pgp\_10\_Efflux}$	$K_{p,br/mu}$
1	23	i.v.	0.568	1.404
4	29	p.o.	2.220	0.237
22	29	p.o.	3.510	0.253
24	23	i.v.	0.903	0.659
32	23	p.o.	4.560	0.397
42	23	p.o.	1.740	1.324
44	23	p.o.	0.826	0.986
45	23	p.o.	11.700	0.129
54	28	p.o.	1.310	0.294
69	28	p.o.	0.717	0.876
72	28	p.o.	1.040	0.614
74	28	p.o.	1.250	0.720
88	28	p.o.	1.400	0.499
93	28	p.o.	1.270	0.312
119	28	i.v.	8.360	0.359
120	28	i.v.	2.080	0.575
137	28	p.o.	0.702	2.154
183	28	p.o.	0.950	0.490
184	28	p.o.	1.430	0.699
199	28	p.o.	1.360	0.866
200	28	p.o.	1.610	0.644
224	20	i.v.	2.260	0.182
233	26	i.p.	1.470	0.208
241	12	p.o.	1.400	1.067
248	42	p.o.	3.140	0.472
249	4	p.o.	20.300	0.197
256	42	p.o.	0.648	0.818
275	23	p.o.	8.200	0.739
296	15	p.o.	0.927	1.665
301	28	p.o.	1.780	1.052
311	15	p.o.	1.390	0.751
318	15	p.o.	1.010	1.589
328	15	p.o.	0.587	1.938
338	15	p.o.	0.991	0.294
346	23	p.o.	1.170	0.657
367	15	p.o.	5.650	0.047
377	15	p.o.	1.390	0.475
385	39	i.v.	1.260	0.533
395	15	p.o.	0.978	0.315
397	15	p.o.	0.627	1.049
443	26	i.p.	3.735	0.137
461	39	s.c.	0.548	1.371
464	39	i.v.	0.668	0.609
605	20	p.o.	0.364	0.742
616	46	p.o.	1.660	0.303
619	23	p.o.	0.640	0.573
623	39	p.o.	0.621	0.791
624	7	p.o.	0.444	2.347
625	27	s.c.	0.708	0.814
626	7	p.o.	0.527	1.136
627	7	p.o.	0.585	1.578
632	27	i.p.	0.638	2.332
633	23	p.o.	0.639	0.798
640	7	p.o.	5.700	0.145
641	7	p.o.	0.534	0.847
642	23	p.o.	1.000	0.834
643	26	i.p.	3.350	0.588
645	7	p.o.	0.721	0.830
654	7	p.o.	0.607	0.549
669	26	i.p.	0.625	2.612
671	28	p.o.	0.545	2.261
26	26	i.p.	2.590	0.263
28	28	p.o.	1.430	0.752
23	23	i.v.	1.330	1.008
23	23	i.v.	15.400	0.112
29	29	p.o.	1.910	0.322
23	23	i.v.	7.840	1.056
23	23	i.v.	2.390	0.088
23	23	i.v.	13.500	0.810
23	23	i.v.	29.400	0.067
23	23	i.v.	0.754	1.307
23	23	i.v.	4.960	0.498
23	23	i.v.	4.490	0.151
23	23	i.v.	6.190	0.538
23	23	p.o.	2.590	0.297
23	23	i.v.	0.425	2.144
23	23	p.o.	2.460	0.376
23	23	p.o.	1.100	0.912
23	23	p.o.	0.658	1.103
23	23	i.v.	7.330	0.214
12	12	p.o.	1.050	2.159
20	20	i.v.	3.810	0.455
12	12	p.o.	1.030	1.558
20	20	i.v.	2.950	0.194
12	12	p.o.	1.600	2.313
12	12	p.o.	1.310	1.168
12	12	p.o.	0.910	0.981
12	12	p.o.	0.794	1.406
12	12	p.o.	0.593	0.988
12	12	p.o.	8.340	0.178
12	12	p.o.	0.758	1.419
23	23	p.o.	1.580	0.896
23	23	p.o.	0.787	0.603
23	23	p.o.	1.380	0.700
23	23	p.o.	6.440	0.327
23	23	p.o.	0.506	0.670
23	23	p.o.	0.627	1.150
23	23	p.o.	2.370	0.646
23	23	p.o.	9.740	0.254
12	12	i.v.	3.950	0.032
51	51	p.o.	0.632	1.017
39	39	s.c.	1.350	0.523
39	39	i.v.	0.508	2.145
39	39	p.o.	1.810	0.356
39	39	p.o.	0.499	0.300
39	39	p.o.	2.540	0.464
39	39	p.o.	4.710	0.038
39	39	p.o.	14.000	0.211
39	39	i.v.	2.080	0.126
39	39	i.v.	0.795	0.896
39	39	p.o.	2.620	0.418
39	39	p.o.	1.640	0.139
51	51	p.o.	0.576	0.707
51	51	p.o.	0.622	1.768
51	51	p.o.	0.697	0.585
51	51	p.o.	0.457	0.754
51	51	p.o.	0.604	0.957
51	51	p.o.	0.526	0.841
26	26	p.o.	4.560	0.130
26	26	p.o.	4.220	0.232
51	51	p.o.	0.550	0.555
39	39	s.c.	0.730	0.877

Table S7: continued

Cpd	Project	Route	$MDCK_{Pgp\_10\_Efflux}$	$K_{p,br/mu}$
843	39	i.v.	1.010	0.208
844	39	i.v.	0.538	0.418
845	39	i.v.	0.841	2.643
846	39	i.v.	0.501	3.467
850	28	p.o.	36.400	0.102
854	26	i.p.	0.612	2.676
855	20	p.o.	14.900	0.168
858	7	p.o.	0.674	0.743
862	7	p.o.	1.500	0.294
863	45	i.v.	0.732	3.750
865	7	p.o.	0.692	1.495
871	26	i.p.	0.772	0.763
874	47	i.v.	0.689	0.757

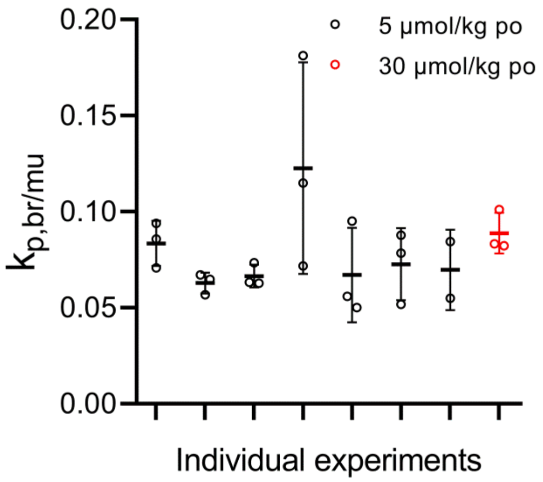

Figure S1. Reproducibility of $K_{p,br/mu}$: 5 µmol/kg (open black circles) or 30 µmol/kg (open red circles) of Compound 5 were administered orally in eight independent experiments. $K_{p,br/mu}$ was determined 0.5 hours after compound administration. Data are mean ± SD, n = 3.

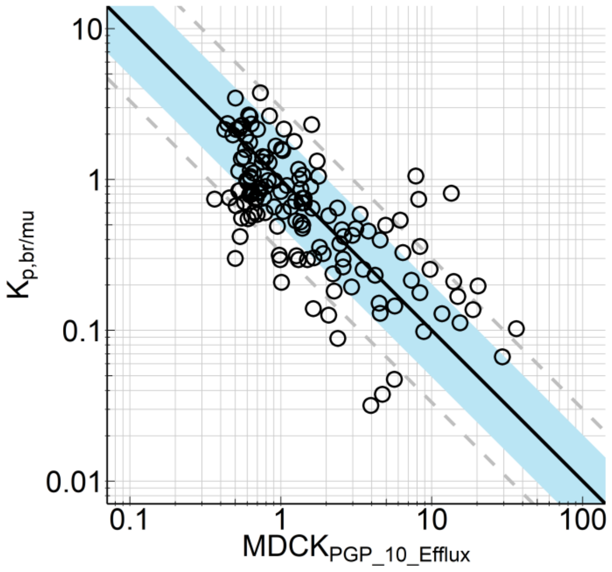

Figure S2. In vitro – in vivo correlation of MDR1 P-gp efflux and mouse $K_{p,br/mu}$. Mouse $K_{p,br/mu}$ is depicted as function of efflux derived from in vitro transporter experiments at 10 µM compound concentration in MDCK-MDR1 cells ($MDCK_{Pgp\_10\_Efflux}$). Correlation analysis was performed with all data depicted in this plot and is characterized by an average food error of 2.2, a Bias of 0.893 and 65.7 % or 84.3 % of data within a 2-fold or 3-fold error. Solid line represents regression, blue area indicates the 2-fold error range, dashed lines are 3-fold error lines. Symbols represent average values (n=2-3 for $K_{p,br/mu}$ and n=2 for in vitro efflux). Data are listed in Supplement Table S7.

Animal experiments in mice were essentially performed as described for rats and under the same regulatory authorization (see 2.2 Animal Studies). Only those aspects are listed here that deviate from the description for rats in the Materials and Methods section of the manuscript. Male mice of the strains C57BL6, CD1, NMRI and Swiss were used. For tissue sampling the animals were anaesthetized with Ketamin/Xylacin. Blood was collected via heart puncture and animals were exsanguinated via the vena cava or the heart. The amount of muscle taken was lower (0.1 to 0.15 g) compared to the taken from rats and the homogenization of muscle was done with a smaller homogenization device (Precellys® kit, 2 ml, CKM). The rest of the procedure, including the homogenization process of the brain, was as described for the rat.
